# N-Terminal Truncated UCH-L1 Prevents Parkinson's Disease Associated Damage

**DOI:** 10.1371/journal.pone.0099654

**Published:** 2014-06-24

**Authors:** Hee-Jung Kim, Hyun Jung Kim, Jae-Eun Jeong, Jeong Yeob Baek, Jaeho Jeong, Sun Kim, Young-Mee Kim, Youhwa Kim, Jin Han Nam, Sue Hee Huh, Jawon Seo, Byung Kwan Jin, Kong-Joo Lee

**Affiliations:** 1 Graduate School of Pharmaceutical Sciences, College of Pharmacy, Ewha Womans University, Seoul, Korea; 2 Department of Biochemistry and Molecular Biology, Neurodegeneration Control Research Center, School of Medicine, Kyung Hee University, Seoul, Korea; National Institutes of Health, United States of America

## Abstract

Ubiquitin C-terminal hydrolase-L1 (UCH-L1) has been proposed as one of the Parkinson's disease (PD) related genes, but the possible molecular connection between UCH-L1 and PD is not well understood. In this study, we discovered an N-terminal 11 amino acid truncated variant UCH-L1 that we called NT-UCH-L1, in mouse brain tissue as well as in NCI-H157 lung cancer and SH-SY5Y neuroblastoma cell lines. *In vivo* experiments and hydrogen-deuterium exchange (HDX) with tandem mass spectrometry (MS) studies showed that NT-UCH-L1 is readily aggregated and degraded, and has more flexible structure than UCH-L1. Post-translational modifications including monoubiquitination and disulfide crosslinking regulate the stability and cellular localization of NT-UCH-L1, as confirmed by mutational and proteomic studies. Stable expression of NT-UCH-L1 decreases cellular ROS levels and protects cells from H_2_O_2_, rotenone and CCCP-induced cell death. NT-UCH-L1-expressing transgenic mice are less susceptible to degeneration of nigrostriatal dopaminergic neurons seen in the MPTP mouse model of PD, in comparison to control animals. These results suggest that NT-UCH-L1 may have the potential to prevent neural damage in diseases like PD.

## Introduction

Ubiquitin C-terminal hydrolase-L1 (UCH-L1) catalyzes the hydrolysis of C-terminal ubiquitin esters and amides. UCH-L1 is highly expressed in metastatic lung cancer [1] and is abundant in brain, comprising 1–2% of total brain protein [2], and is a major component of the protein aggregates called Lewy bodies found in the brains of PD patients [3]. Also, a mutant of UCH-L1, I93M (Ile93 to Met), was shown to cause a type of autosomal dominant PD in one German family [4]. These disparate observations have led to a suggestion that UCH-L1 may be a PD related gene. However, the molecular connection between UCH-L1 and PD was not fully established.

Mutations, environmental stresses, and aging cause protein denaturation, rendering them aggregation-prone forms [5]. Chaperones play roles in refolding the denatured proteins or for clearing these in ubiquitin-proteasome system and in autophagy [6]–[8]. However, when these defense systems fail to repair, protein aggregates accumulate and induce cell death [5]. Parkinson's disease (PD) is known to associated with formation of protein aggregates and Lewy bodies as hallmarks of PD [9], [10]. Although there is evidence that protein aggregates are toxic to cells [11], [12], it is not a necessary and sufficient condition to develop PD in human patients and animal models [13]–[15]. In fact, Lewy bodies have been suggested to have neuroprotective effect [16]–[18]. For example, α-synuclein, another PD causing protein, is known to form various oligomeric structures, which show both toxicities and protective effects on cells [19], [20].

Post-translational modifications of proteins and alternative splicing can change biochemical properties including solubility of a protein. Phosphorylation, ubiquitination, and truncation affect aggregation behavior of α-synuclein [21]. In the case of UCH-L1, carbonylation and modification by cyclopentenone prostaglandin, decrease its solubility supporting its possible relationship with PD [22], [23]. Monoubiquitination of UCH-L1 was also reported to restrict its hydrolase activity [24], but its effect on the solubility of UCH-L1 has not been studied. In general, polyubiquitination is required for proteasomal degradation of a protein while monoubiquitination enables its participation in DNA repair, histone regulation, gene expression, and receptor endocytosis [25].

The discovery that 1-methyl-4-phenyl-1,2,3,6-tetrahydrodropyridine (MPTP) infusion causes Parkinsonism by selective inhibition of mitochondrial complex-1, raised the possibility that mitochondrial dysfunction is at the heart of PD. Mitochondrial dysfunction has been commonly observed in autopsied PD brain tissues [26]. Most PD-related gene products are found in mitochondria [27]–[29] and overexpression, deletion or mutation of several familial PD-related gene products (α-synuclein, parkin, PINK1, and LRRK2) affect mitochondrial function, integrity, and susceptibility to mitochondrial toxins [30]–[33]. However, the effects of UCH-L1 on mitochondria have not been studied.

Oxidative stress has been shown to be the cause of nigrostriatal dopaminergic neuron loss in PD patients and in the MPTP mice model of PD [34]. Toxins such as MPTP, rotenone, 1,1′-dimethyl-4,4′-bipyridinium dichloride (paraquat), and 6-hydroxydopamine (6-OHDA), which induce PD like symptoms in mice are all oxidative stress inducers [35]–[38]. Cysteine thiol group (-SH) of proteins are susceptible to oxidative stress and are readily oxidized to disulfide, sulfenic acid, sulfinic acid and sulfonic acid. Disulfide crosslinking resulting in protein aggregation, has been demonstrated in the development of various diseases [39], [40].

In this study we identified a variant of UCH-L1 lacking N-terminal 11 amino acids designated as NT-UCH-L1, and compared it to UCH-L1 by physical, chemical, and proteomic approaches with the goal of understanding their possible relation to PD. We found that NT-UCH-L1 is aggregation prone and is localized in mitochondria, which are regulated by monoubiquitination. Furthermore, NT-UCH-L1 was found to have a protective role in the PD model *in vitro* and *in vivo*.

## Experimental Procedures

### Cell culture

Human NCI-H157, SH-SY5Y, and HeLa cells were maintained respectively in RPMI 1640, dulbecco's modified eagle medium and minimum essential medium supplemented with 10% fetal bovine serum, 100 µM/mL of streptomycin and 100 units/mL of penicillin G (all from Invitrogen).

### Antibodies

The sources of antibodies used in this study were as follows: Monoclonal anti-myc antibody from Millipore, polyclonal anti-UCH-L1 and anti-ubiquitin antibody from Chemicon, anti-flag antibody (M2) from Sigma, anti-tubulin antibody from Santa Cruz Biotechnology, Alexa Fluor 488 goat anti-mouse antibody from Molecular Probes. Polyclonal antibodies against N-terminal peptide of UCH-L1 were generated in rabbits and characterized by Abclon (Korea).

### 2D gel electrophoresis

This was performed as described previously [41].

### Establishment of UCH-L1 expressing stable cell lines

The lentiviral vector for expressing wild type UCH-L1 and NT-UCH-L1 was constructed by inserting PCR gene fragment into the XbaI–EcoRV site of LentiM1.4 vector. Pseudotyped lentiviruses were produced and used for stable expression of UCH-L1s as described earlier [42].

### Measurement of ubiquitin C-terminal hydrolase activity

Ubiquitin C-terminal hydrolase activity was measured using the Ub-AMC (AG scientific) as substrate [1]. Five nM of GST-UCH-L1 or GST-NT-UCH-L1 were incubated with 0–1000 nM of Ub-AMC and the release of free AMC monitored at 460 nm.

### Hydrogen/Deuterium exchange (HDX) mass spectrometry

UCH-L1 and NT-UCH-L1 (about 1 µg/µL) were diluted 10-fold with D_2_O and maintained at 25°C for several time scales. The labeling reaction was quenched by 5 mM tris(2-carboxyethyl)phosphine (TCEP), pH 2.3. For peptic digestion, porcine pepsin (1 µg/µL) was added to each quenched protein sample and incubated at 0°C for 3 min before injection [43], [44]. Peptic peptides were desalted and separated as previously described [41]. The autosampler chamber was set at 5°C. The trap, analytical column and all tubing were immersed in an ice bath to minimize deuterium back-exchange. Both mobile phase bottles containing 0.1% formic acid were placed on ice. Gradient chromatography was performed at a flow rate 0.6 µL/min and was sprayed on line to nanoAcquity™/ESI/MS (SYNAPT™ HDMS™, Waters). All mass spectral measurements were taken at: capillary voltage 2.5 kV, cone voltage 35 V, extraction cone voltage 4.0 V, and source temperature 80°C. TOF mode scan was performed in the range of *m*/*z* 300–1500 with scan time of 1 s.

### Mass spectrometry

The gel spots of proteins were destained and digested with trypsin and the resulting peptides were analyzed by nanoAcquity UPLC/ESI/MS (SYNAPT HDMS, Waters) as described previously [41], [45], [46]. The peptides were separated using a C18 reversed-phase 75 µm i.d. x 150 mm analytical column (1.7 µm particle size, BEH130 C18, Waters) with an integrated electrospray ionization PicoTip (±10 µm, New Objective). Seven µL of peptide mixtures were dissolved in buffer A (Water/formic acid; 100∶0.1, v/v), injected on the column and eluted by a linear gradient of 5–80% buffer B (acetonitril/formic acid; 100∶0.1, v/v) over 120 min. Samples were desalted on line prior to separation using a trap column cartridge (ID 180 µm x 20 mm, Symmetry C18, Waters). Initially, the flow rate was set to 300 nL/min and a capillary voltage of 2.5 keV was applied to the LC mobile phase before spray. Chromatography was performed on line to mass spectrometer. MS parameters for efficient data-dependent acquisition were: intensity of >10 and 3 components to be switched from MS to MS/MS analysis.

The individual MS/MS spectra acquired from each of the precursors within a single LC run were combined, smoothed, deisotoped and centroided using the Micromass ProteinLynx Global Server (PLGSTM) 2.1 data processing software and output, as a single MASCOT-searchable peak list (.pkl) file. The peak list files were used to query the SwissProt database using the MASCOT (global search engine), with the following parameters: peptide mass tolerance, 0.2 Da; MS/MS ion mass tolerance, 0.2 Da; allowing up to 1 missed trypsin cleavage site, considering variable modifications, such as acetylation, deamidation, pyro-glu (N-term E, Q), oxidation, formylation, phosphorylation, carbamidomethyl and cysteine propionamide but not fixed modifications; enzyme limited to trypsin; and toxonomy limited to mouse. All reported assignments were verified by automatic and manual interpretations of spectra from MASCOT.

### Preparation of soluble and insoluble fractions of cell lysates

Soluble and insoluble fractions of cell lysates were prepared as previously described [47]. Briefly, cells from 100 mm cell culture plates were lysed in 500 µL RIPA buffer (50 mM Tris-Cl, pH 8.0, 150 mM NaCl, 1% (v/v) NP-40, 0.5% sodium deoxycholate and 0.1% SDS, protease inhibitor cocktail (Sigma), 10 mM NEM) by passing 10 times through a 26.5-gauge needle. After centrifugation at 12,000 x g for 20 min, pellets were resuspended in 200 µL of gel sample buffer (62.5 mM Tris-Cl, pH 6.8, 10% (w/v) glycerol, 5% β-mercaptoethanol, 2.3% SDS). The supernatant was mixed with same volume of gel sample buffer and 20 µL of each fraction were resolved in 10% SDS-PAGE.

### Immunoprecipitation

Cells or mouse brain tissues were lysed with immunoprecipitation buffer (50 mM Tris-Cl, pH 7.4, 50 mM NaCl, 0.5 mM EDTA, 1 mM PMSF, 5 µg/mL aprotinin, 10 µg/mL leupeptin, 10 µg/mL pepstatin A, 1 mM Na_3_VO_4_, 0.5% NP-40). Lysates were centrifuged at 14,000 rpm for 10 min and the supernatant was incubated for 3 h at 4°C with anti-flag or anti-myc antibody cross-linked to protein G sepharose beads. Beads were washed 3 times with immunoprecipitation buffer, bound proteins were eluted by gel sample buffer and separated on SDS-PAGE.

### Confocal microscopy

Cells were grown on the SecureslipTM cell culture cover slip (Gracebiolab). Cells were then incubated with 250 nM of MitoTracker Red 580 in HBSS at 37°C for 30 min and fixed with 4% paraformaldehyde for 10 min at room temperature and permeabilization was done with 0.1% Triton X-100 in HBSS for 15 min at room temperature. The cells were treated with blocking solution (3% BSA, 0.2% Tween 20 and 0.2% gelatin in PBS) for 1 h at room temperature and incubated with monoclonal anti-myc antibody, diluted 1∶250 in HBSS containing 1% BSA and 1% sucrose for 1 h at 37°C. After three washes with PBS, the cells were incubated with Alexa Fluor 488 conjugated goat anti mouse IgG diluted 1∶50 for 1 h at 37°C. After three washes, the cells were analyzed under Zeiss LSM 510 META confocal microscope.

### Preparation of mitochondrial fractions

Mitochondrial fractions of the cells were prepared as described previously [48]. Cells were suspended in 200 µL of 10 mM Tris-Cl, pH 7.5, supplemented with protease inhibitor cocktail and 10 mM NEM and passed through a 26.5-gauge needle 20 times and 40 µL of ice-cold 1.5 M sucrose solution was added and centrifuged at 600 x g for 10 min, the supernatants were centrifuged at 14,000 x g for 10 min and mixed with the same volume of gel sample buffer. The pellets containing mitochondrial fraction were washed with 50 µL of 10 mM Tris-Cl, pH 7.5 twice and lysed in 30 µL of gel sample buffer. Same volumes of the supernatant and gel sample buffer mixture and mitochondrial fraction in gel sample buffer, were resolved in 10% SDS-PAGE.

### Measurement of ROS

Cellular ROS were measured using CM-H_2_DCFDA (Invitrogen) following manufacturer's instructions. Briefly, trypsinized cells were washed and resuspended in 0.5 mL of HBSS; 3 µL of 0.5 mM CM-H_2_DCFDA was added and incubated at 37°C for 15 min. After brief wash with HBSS, the cells were analyzed with FACS Calibur flow cytometer (BD Biosciences).

### Real-time cell analysis

Five thousand HeLa cells were plated in a 96 well E-plate for the real-time cell analyzer, xCELLigence (Roche Applied Science). For H_2_O_2_ treatment, cells were treated with H_2_O_2_ for 1 h before plating in the E-plate. After 24 h, the cells were treated with rotenone or CCCP and cell growth was monitored by measuring electrical impedance every 30 min.

### Generation of hNT-UCH-L1 expressing transgenic mice

We generated transgenes by cloning the hNT-UCH-L1-myc DNA under the control of CAG promoter in pCAGEN vector (Addgene). The plasmid was linearized by digestion with ScaI and BamHI and microinjected into the pronuclei of newly fertilized C57BL/6N mouse eggs (Macrogen). Germline transmission of NT-UCH-L1 was obtained in five independent NT-Tg lines and the levels of transgenic mRNA were assessed by quantitative RT-PCR. cDNAs from mouse whole brain were prepared using RNA extraction kit (Qiagen) and SuperScript III (Life Technologies) reverse transcriptase. For quantitative RT-PCR, specific primers for the transgene (forward: 5′-TGCTGAACAAAGTGCTGTCC-3′; reverse: 5′-AGCCCAGAGACTCCTCTTCC-3′) were used. The offsprings were screened with specific primers (forward: 5′-CTTTGTCCCAAATCTGGCGGA-3′; reverse: 5′-TGGCCACTGCGTGAATAAGTC-3′). All experiments were performed in accordance with approved animal protocols and guidelines established by Ewha Womans University [2011-01-025] and Kyung Hee University [KHUASP(SE)-10-030].

### MPTP treatment and analysis of hNT-UCH-L1 transgenic mice

All experiments were conducted with eight-week-old male C57BL/6N mice (23–25 g) maintained in a room at 20–22°C on 12 h light/dark cycle with food and water available *ad libitum*. As described previously [49]–[51], mice were administered four i.p. injections of MPTP (20 mg/kg, free base; Sigma-Aldrich) dissolved in PBS at 2 h intervals, and were transcardially perfused and fixed with 4% paraformaldehyde dissolved in 0.1 M phosphate buffer (PB). Brain tissues were cut into 30-µm-thick coronal sections and processed for immunostaining, as described [49], [50]. In brief, the brain sections were rinsed and incubated overnight at room temperature with primary antibodies, anti-TH, at 1∶2000 dilution; Pel-Freez Biologicals) for DA neurons and anti-OX-42 (CD11b, at 1∶400 dilution; Serotec) for microglia. On the following day, the sections were rinsed, incubated with the appropriate biotinylated secondary antibody and processed with an avidin-biotin complex kit (Vectastain ABC kit; Vector Laboratories). Bound antibody was visualized by treatment with diaminobenzidine-HCl (Sigma-Aldrich) and hydrogen peroxide (Sigma-Aldrich). The diaminobenzidine reaction was terminated by rinsing the tissues in 0.1 M PB. Labeled tissue sections were mounted on gelatin-coated slides and analyzed under a bright-field microscope (Nikon).

### Stereological cell counts

As described previously [49], [50], the total number of TH-positive neurons was counted in the various animal groups at 7 days post injection (MPTP or PBS) using the optical fractionators method performed on an Olympus Computer Assisted Stereological Toolbox system version 2.1.4 (Olympus Denmark). The counting was performed using a 100X oil objective. The total number of neurons was estimated using the optical fractionator equation [52].

### Densitometric analysis

As described previously [49], [50], the OD of TH-positive fiber in STR was examined at 35 original magnification using the IMAGE PRO PLUS system (Version 4.0; Media Cybernetics) on a computer attached to a light microscope (Zeiss Axioskop) interfaced with a CCD video camera (Kodak Mega Plus model 1.4 I; Kodak). To control variations in background illumination, the average of background density readings from the corpus callosum was subtracted from that of density readings of the STR for each section. For each animal, the average of all sections was calculated separately before data were statistically processed.

## Results

### Discovery of N-terminal truncated UCH-L1 (NT-UCH-L1)

**Figure 1:**
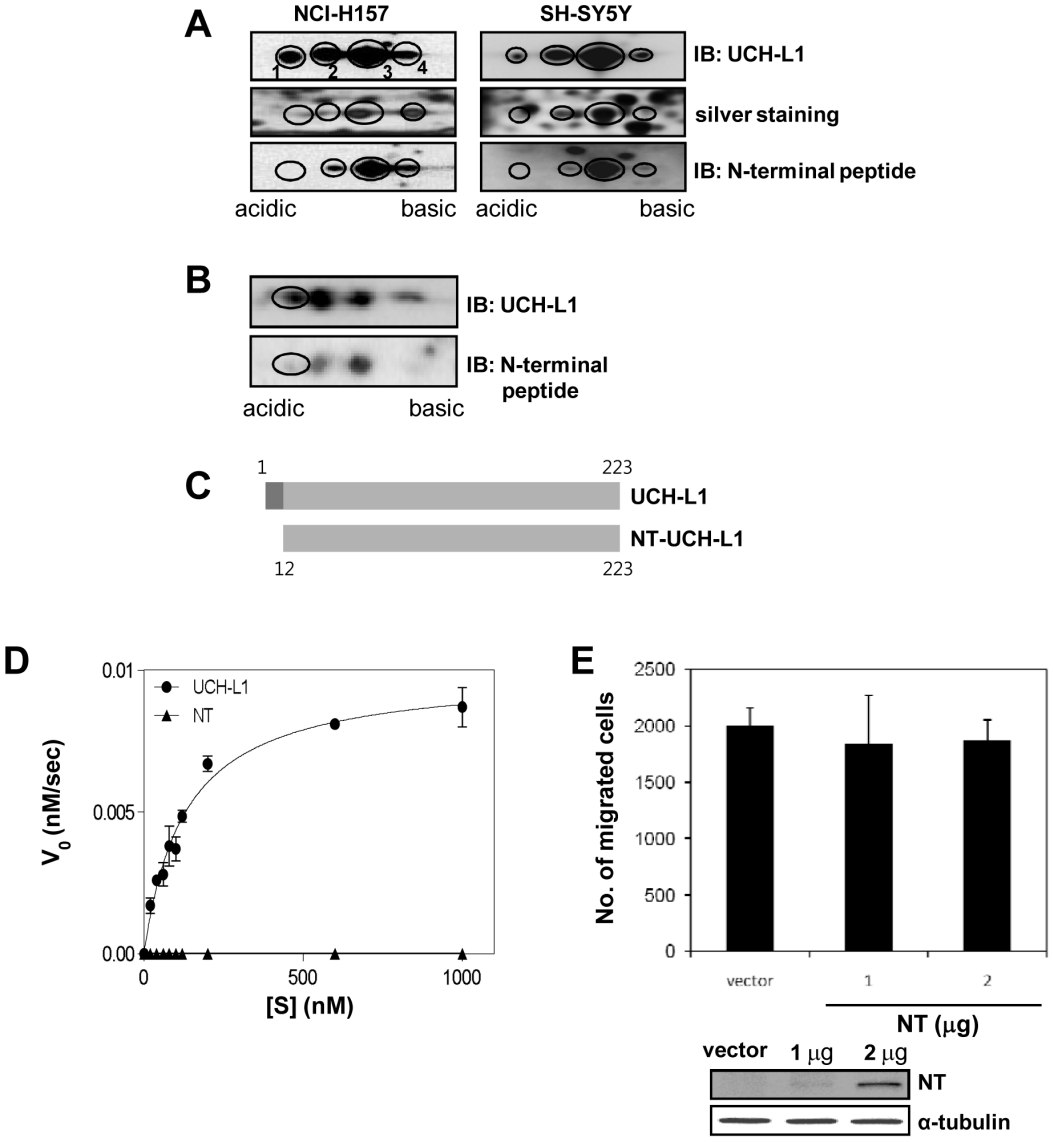
Discovery of NT-UCH-L1 in human lung cancer and neuroblastoma cell lines and its enzymatic and structural differences from UCH-L1. (A) NCI-H157 (left panels) and SH-SY5Y (right panels) cells were analyzed using 2D-PAGE and visualized by Western blot analysis using anti-UCH-L1 (top panels), anti-N-terminal peptide (against the peptide, ^1^MQLKPMEINPE^11^) antibodies (lower panels), or silver staining (middle panels). Four spots representing UCH-L1 are numbered from 1 to 4. (B) Mouse whole brain tissue was analyzed using 2D-PAGE and visualized by Western blot analysis using anti-UCH-L1 (top panel) and anti-N-terminal peptide antibodies (lower panel). The location of NT was marked by a circle in each panel. (C) A diagram of UCH-L1 and N-terminal 11 amino acid truncated NT-UCH-L1. (D) Ubiquitin hydrolase activities of GST-UCH-L1 and GST-NT-UCH-L1 were measured using Ub-AMC as a substrate. 5 nM of GST-UCH-L1 or GST-NT-UCH-L1 was incubated with 0–1000 nM of Ub-AMC and monitored the release of free AMC at 460 nm. (E) HeLa cells transiently expressing NT-UCH-L1 were subjected to migration assay using transwell coated with Matrigel™. After 24 h of seeding, the number of migrated cells in the lower chamber was counted. The expression of NT-UCH-L1 in HeLa cells were verified by Western blot analysis using anti-myc antibody, and anti-α-tubulin antibody as a loading control.

We screened for variants of UCH-L1, several of which are shown in Fig. S1A in human lung cancer cell line, NCI-H157, and neuroblastoma cell line, SH-SY5Y. We detected four UCH-L1 spots by immunoblotted 2D-PAGE (Fig. 1A, left top panel). The spots from the corresponding silver stained gels (Fig. 1A, left middle panel) were characterized using mass spectrometry (MS). We found that spot 1 was missing the peptide ^1^MQLKPMEINPEMLNK^15^ (1815.9144 Da) of UCH-L1, but this was replaced by ^12^MLNK^15^ (505.2905 Da) (Fig. S1B). The peptide, missing in spot 1, was present in spot 3, and both spots had similar molecular weights about 25 kDa, suggesting that spot 3 was full length UCH-L1 while spot 1 is a truncated UCH-L1 missing an N-terminal peptide (NT-UCH-L1). To confirm this, we raised polyclonal antibodies (anti-N-terminal peptide) against the peptide, ^1^MQLKPMEINPE^11^ in rabbit, and examined their reactivity with the four spots. The antibodies did not react with spot 1 which lacks the peptide, but reacted with spots 2, 3, and 4 (Fig. 1A left bottom panel). NT-UCH-L1 was also detected in human neuroblastoma cell line, SH-SY5Y (Fig. 1A, right panels) and mouse whole brain tissue (Fig. 1B). The deleted peptide of NT-UCH-L1, corresponds to exon 1 of the *UCH-L1* gene (*PARK5*) (Fig. 1C). The exon 1 deleted form is an mRNA registered in NCBI nucleotide database under the name, protein gene product 9.5 (PGP9.5, accession number X04741). Given the structure of its mRNA, NT-UCH-L1 appears to be formed by translation from alternatively spliced mRNA.

### Biochemical and Structural comparison of NT-UCH-L1 and UCH-L1

NT-UCH-L1 did not exhibit the ubiquitin hydrolase activity of UCH-L1 (Fig. 1D and S1C). Also, NT-UCH-L1, unlike UCH-L1, did not increase the invasiveness of cancer cells as reported previously (Fig. 1E and S1D) [1]. Thus NT-UCH-L1 is biochemically and biologically distinct from full length UCH-L1.

**Figure 2:**
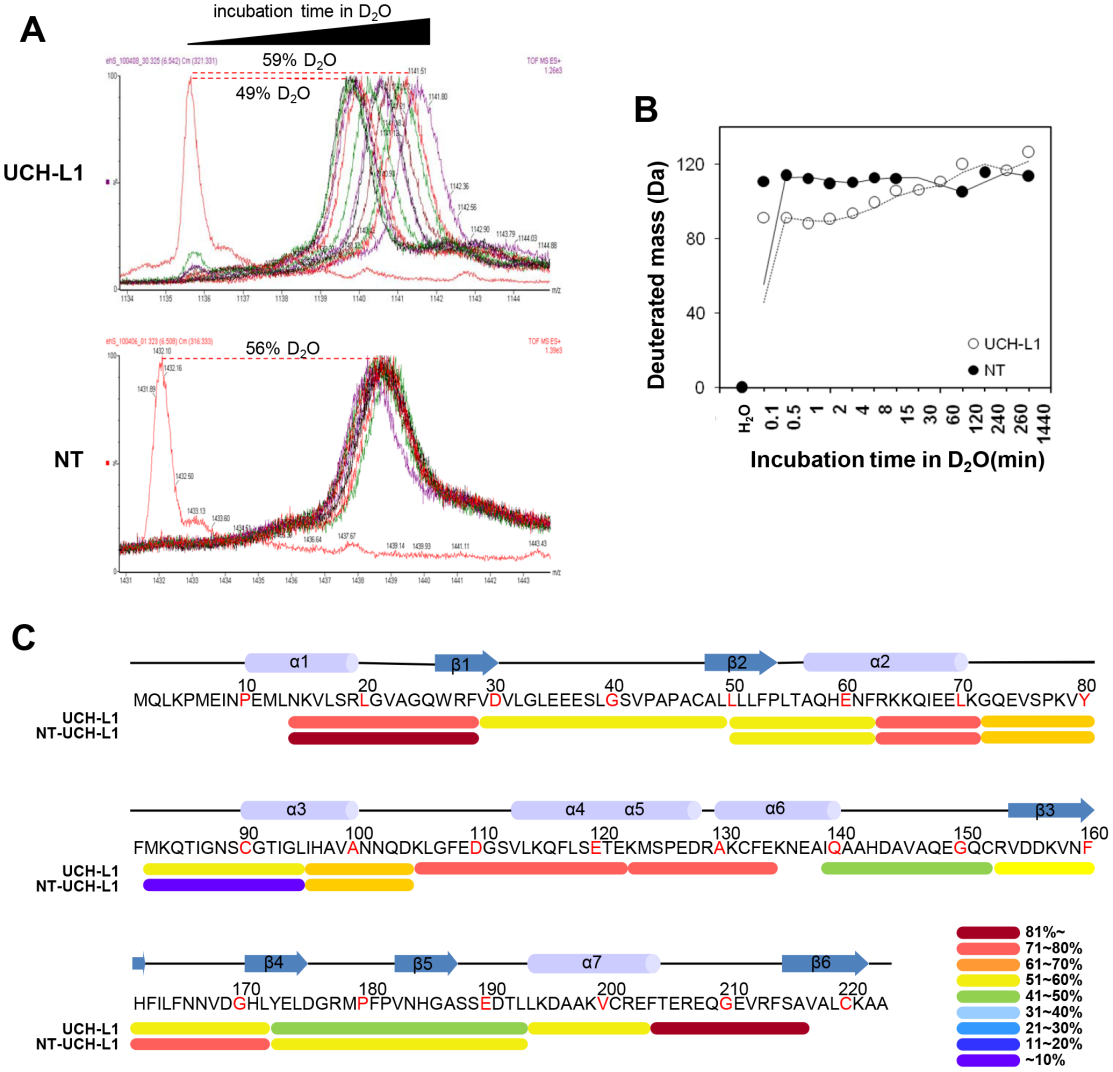
Structural differences between UCH-L1 and NT-UCH-L1. (A, B) Purified UCH-L1 and NT-UCH-L1 were subjected to HDX studies. UCH-L1 and NT-UCH-L1 were incubated with D_2_O exchange buffer at 25°C for the indicated times and analyzed using nanoAcquity™/ESI/MS. HDX spectra of UCH-L1 and NT-UCH-L1 and deuterium exchange rates were represented by % exchange (A) and mass increase (B). (C) Recombinant UCH-L1 and NT-UCH-L1 were subjected to HDX studies. Proteins were incubated with D_2_O exchange buffer at 25°C for 30 min, digested with trypsin and analyzed using nanoAcquity™/ESI/MS. Deuterium exchange rates were represented by % exchange and colored accordingly.

In order to understand the structural basis of these differences, we employed mass spectrometry with hydrogen/deuterium exchange (HDX) in which residues exposed to surface more readily exchange deuterium allowing their mass increases to be detected. HDX occurred slowly in UCH-L1, taking up to 16 h to complete, while in NT-UCH-L1, this exchange was completed in just about 2 min (Fig. 2A and B). This indicates that NT-UCH-L1 has a more flexible structure than UCH-L1. The flexible regions were identified by analyzing HDX in pepsin-digested peptides (Fig. 2C). More deuterium exchange occurred in the N- and C-termini of NT-UCH-L1 than of UCH-L1, and the peptide containing active site cysteine (^82^MKQTIGNS**C**GTIGL^95^) in NT-UCH-L1 exchanged less deuterium than in UCH-L1. These results indicate that truncation of N-terminal 11 amino acids induces remarkable structural changes.

### NT-UCH-L1 is monoubiquitinated at either Lys15 or Lys157

**Figure 3:**
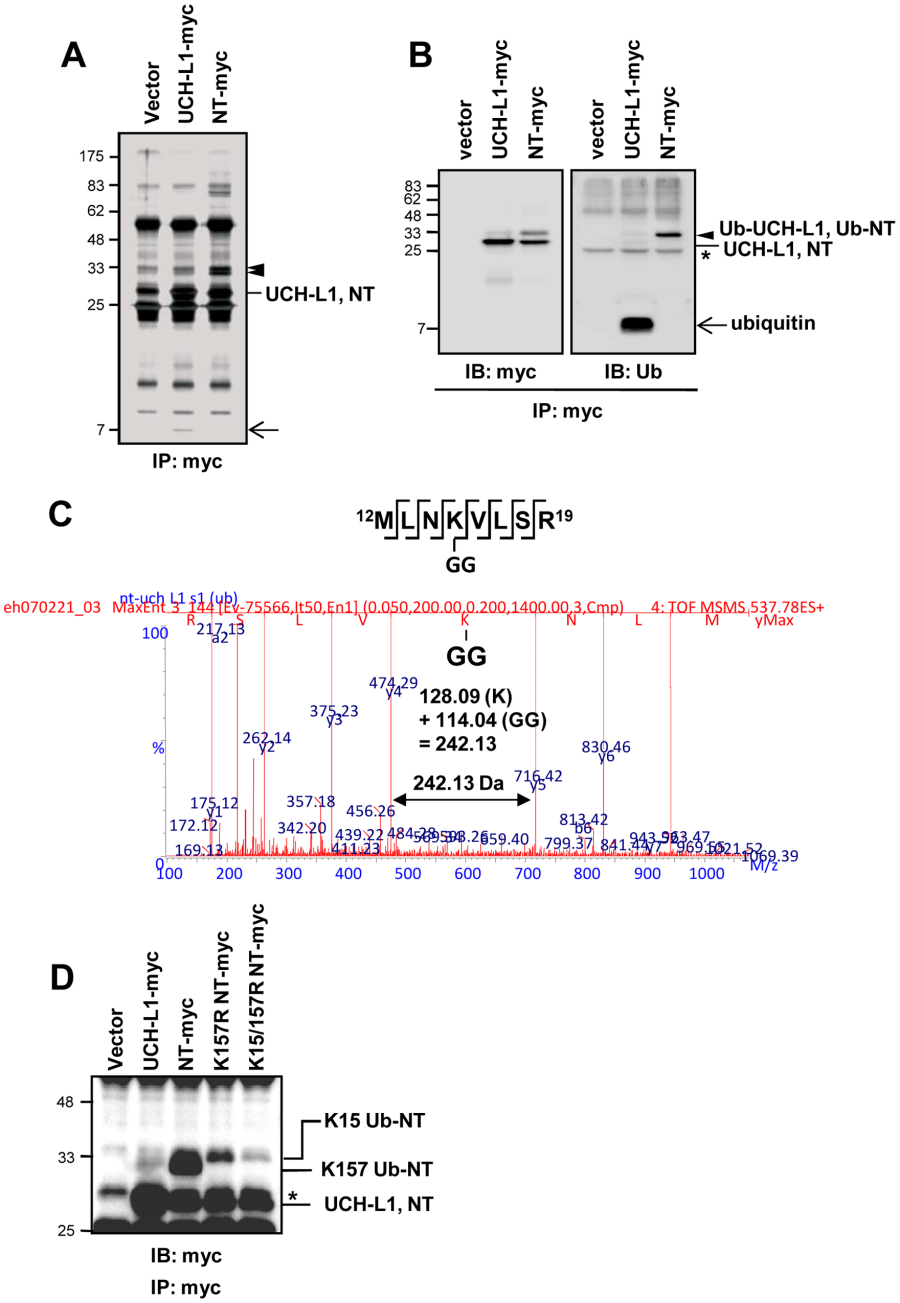
Monoubiquitination of NT-UCH-L1. (A, B) NCI-H157 cells transiently transfected with empty vector, pcDNA3.1 UCH-L1-myc or pcDNA3.1 NT-UCH-L1-myc were subjected to immunoprecipitation using anti-myc antibody. Beads bound proteins were resolved in 13% SDS-PAGE and visualized by silver staining (A) and Western blot analysis using anti-myc and anti-ubiquitin antibodies (B). *, non-specific bands. (C) A tandem MS spectrum of a peptide of NT-UCH-L1 containing Lys15. It was sequenced using nanoLC-ESI-q-TOF tandem MS and the GlyGly ubiquitin C-terminus fragment on Lys15 was detected. (D) HeLa cells stably expressing UCH-L1-myc, NT-UCH-L1-myc and K15/157R NT-UCH-L1-myc were examined. Cells were immunostained with anti-myc antibody to confirm the monoubiquitination sites.

UCH-L1 recruited free ubiquitin resulting in high cellular free ubiquitin levels [53]. We therefore examined possible differences between NT-UCH-L1 and UCH-L1, in binding free ubiquitin. NCI-H157 cells transiently expressing UCH-L1-myc or NT-UCH-L1-myc were immunoprecipitated and the resulting precipitates were analyzed (Fig. 3A). We detected several bands including UCH-L1-myc and NT-UCH-L1-myc in silver stained gel. The unknown bands were identified by peptide sequencing with MS/MS. One band of about 7 kDa, co-precipitating with UCH-L1, was identified as previously shown, as ubiquitin non-covalently bound to UCH-L1 (Fig. 3A arrow, 3B right panel arrow), in agreement with previous studies [53]. Two bands co-precipitating with NT-UCH-L1 (Fig. 3A arrow heads) were identified as mixtures of NT-UCH-L1 and ubiquitin. We found that the molecular weight difference between NT-UCH-L1-myc and the two bands was close to the molecular weight of one ubiquitin. We performed Western blot analysis with anti-myc (Fig. 3B left panel) and anti-ubiquitin (Fig. 3B right panel) antibodies and confirmed the upper band as monoubiquitinated NT-UCH-L1 (Ub-NT-UCH-L1). When NCI-H157 cells were analyzed in 2D-PAGE and blotted with anti-UCH-L1 or anti-N-terminal peptide antibody, we detected a shifted NT-UCH-L1 spot possibly endogenous ubiquitinated NT-UCH-L1 (Fig. S2 arrow).

We identified the sites of monoubiquitinated lysine residue(s) in NT-UCH-L1, employing both peptide sequencing with MS/MS and mutation studies. We analyzed peptides of immunoprecipitated and trypsinized NT-UCH-L1 by MS/MS and detected Lys15 having a mass increase 114 Da which is equivalent to C-terminal Gly-Gly of attached ubiquitin. This indicates that the candidate site of ubiquitination is Lys15 (Fig. 3C). We constructed two mutants of NT-UCH-L1, K157R (a previously known monoubiquitination site of UCH-L1) [24] and K15/157R, and examined monoubiquitination of these mutants (Fig. 3D). Monoubiquitination of NT-UCH-L1 was reduced in K157R NT-UCH-L1 and was mostly absent in K15/157R NT-UCH-L1 mutants. These results suggest that there are two populations of Ub-NT-UCH-L1, one monoubiquitinated at K15 and the other at K157, not both.

### Ub-NT-UCH-L1 localizes in cytosol while unubiquitinated-NT-UCH-L1 localizes in mitochondria

**Figure 4:**
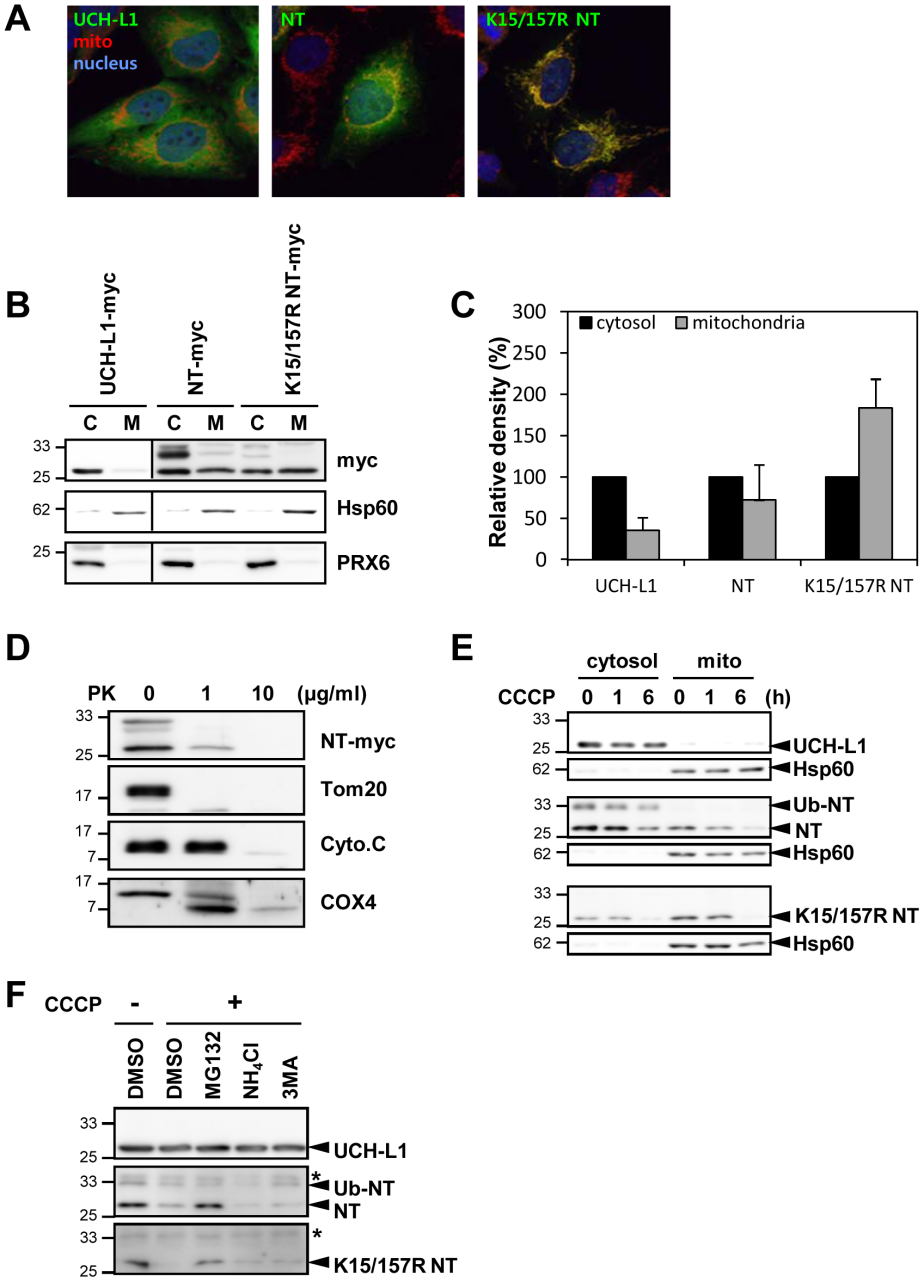
Localization of NT-UCH-L1 in mitochondria. (A) HeLa cells stably expressing UCH-L1-myc, NT-UCH-L1-myc and K15/157R NT-UCH-L1-myc were stained with anti-myc and Alexa Fluor 488 secondary antibody (green). Mitochondria and nucleus were stained with Mitotracker (red) and DAPI (blue), respectively. Cells were visualized by confocal microscopy. (B) Cells were fractionated into cytosolic and mitochondrial fractionations. UCH-L1s were immunoblotted using anti-myc antibody and Hsp60 and PRX6, a mitochondrial and cytosolic marker protein, respectively, were immunoblotted using anti-Hsp60 and anti-PRX6 antibodies. The relative distribution of UCH-L1-myc, NT-UCH-L1-myc and K15/157R NT-UCH-L1-myc in the cytosolic and mitochondrial fractions were quantified by measuring their density (C). The mean ± s.d. of three independent experiments is shown. (D) Mitochondrial fractions of HeLa cells expressing NT-UCH-L1 were incubated with various concentrations of proteinase K for 1 h at 50°C and immunoblotted with anti-myc, Tom20, cytochrome C and COX4 antibodies. (E) Cells were treated 10((M CCCP for 0, 1, or 6 h and fractionated into cytosolic and mitochondrial fractionations. UCH-L1s were immunoblotted using anti-myc antibody and Hsp60, a mitochondrial marker protein, was immunoblotted using anti-Hsp60 antibody. (F) Cells were treated with 10((M CCCP in combination with 10((M MG132 (an inhibitor of proteasomal degradation), 10 mM NH4Cl (an inhibitor of lysosomal degradation) or 10 mM 3MA (an inhibitor of autophagy). UCH-L1s were immunoblotted using anti-myc antibody.

The focus of PD research has been on mitochondrial functions and its respiratory activities. The presence of PD-related gene products in autopsied PD brain tissues and in mitochondria, underscores their effect on mitochondrial functions [26]–[33]. We therefore compared the subcellular localizations of UCH-L1 and NT-UCH-L1. We used HeLa cells because the expression of endogenous UCH-L1 in these cells was blocked by methylation of the *UCH-L1* gene [54]. In confocal microscopy, the majority of UCH-L1-myc localizedin the cytosol, while NT-UCH-L1-myc localized in both cytosol and mitochondria (Fig. 4A). Moreover, ubiquitination-free K15/157R NT-UCH-L1 localized primarily in the mitochondria. This suggests that monoubiquitination governs subcellular localization of NT-UCH-L1. These localizations were corroborated by Western blot studies of crude fractionated cytoplasm and mitochondria. Whereas full length UCH-L1 and the ubiquitinated species of NT-UCH-L1 were found predominantly in the cytosol, the non-ubiquitinated NT-UCH-L1 and K15/157R NT-UCH-L1 were found in both the cytosol and mitochondrial fractions, but more predominantly in mitochondria (Fig. 4B and 4C). It is possible that the binding of K15/157R NT-UCH-L1 with mitochondrial outer membrane might be weak and results in its partial cytosolic localization in mitochondrial fractionation assay. Although some part of UCH-L1 was monoubiquitinated as shown in Fig. 3B, most of UCH-L1 and its monoubiquitination free form localized in cytosol (Fig. S3A). Monoubiquitination on UCH-L1 did not seem to determine its subcellular localization.

To further dissect the location of NT-UCH-L1 in mitochondria, we incubated the mitochondrial fractions with varying concentrations of proteinase K and performed Western blot analysis with antibodies specific for Tom20, cytochrome C, and COX-4, markers of the outer membrane, intermembrane space, and inner membrane of mitochondria, respectively (Fig. 4D). The proteolytic degradation pattern of NT-UCH-L1 was similar to that of Tom20 suggesting that NT-UCH-L1 localized in the outer membrane of mitochondria.

Since Parkin, another PD related protein, is recruited to mitochondria and degraded via mitotophagy in response to mitochondrial damage [55], we investigated whether mitochondrial localization of NT-UCH-L1 is related to *m*-chlorophenylhydrazone (CCCP) induced mitochondrial damage. We did not detect any increase of NT-UCH-L1 in mitochondria after CCCP treatment (Fig. 4E). Rather, NT-UCH-L1 in mitochondrial fraction decreased. When HeLa cells were treated with CCCP in combination with MG132, NH_4_Cl, and 3MA, inhibitors of proteasomal, lysosomal, and autophagic degradation, respectively, both NT-UCH-L1 and its K15/157R mutant were protected from degradation by MG132 treatment suggesting proteasomal degradation of NT-UCH-L1 (Fig. 4F). That means NT-UCH-L1's degradation pathway is clearly distinguishable from parkin involved mitophagy.

### NT-UCH-L1 turns over faster than UCH-L1

**Figure 5:**
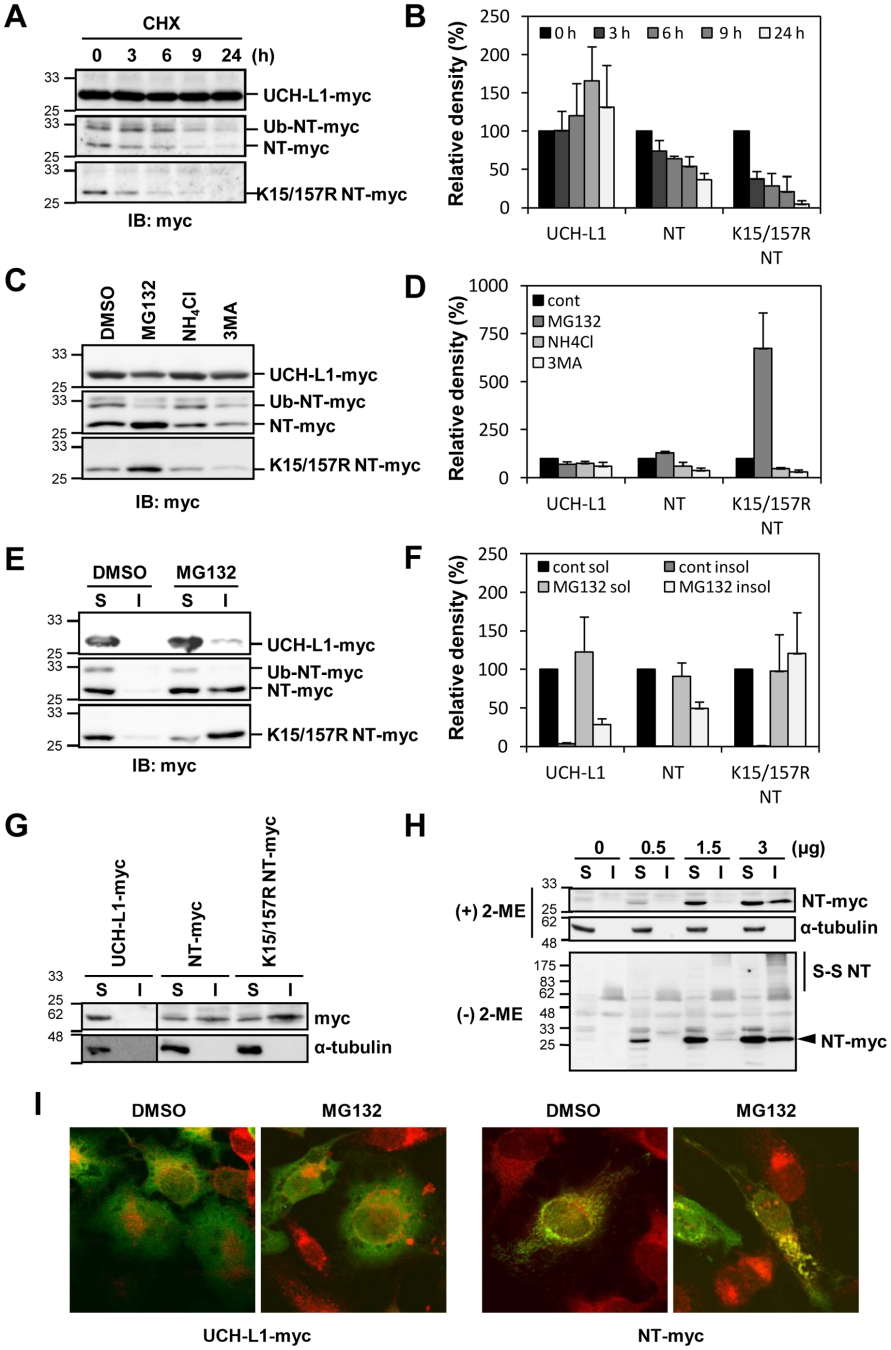
Degradation and aggregation-proneness of NT-UCH-L1. HeLa cells stably expressing UCH-L1-myc, NT-UCH-L1-myc and K15/157R NT-UCH-L1-myc were examined. (A, B) Cells were treated with 10 µg/mL cycloheximide for the indicated times and immunoblotted using anti-myc antibody (A) and the bands were quantified (B). (C, D) Cells were treated with DMSO, 10 µM MG132, 10 mM NH_4_Cl or 10 mM 3MA 16 h and immunoblotted using anti-myc antibody (C) and the bands were quantified (D). (E, F) Cells were treated with DMSO or 10 µM MG132 for 9 h and divided into soluble and insoluble fractions. Samples were immunoblotted using anti-myc antibody (E) and the bands were quantified (F). Quantitative analysis was done and the mean ± s.d. of three independent experiments is shown. (G) SN4741 cells were transiently transfected with pcDNA3.1 UCH-L1-myc, pcDNA3.1 NT-UCH-L1-myc, and pcDNA3.1 K15/157R NT-UCH-L1-myc and fractionated into soluble and insoluble fractions. Fractions were immunoblotted using anti-myc and anti-α-tubulin. Due to the higher expression level of UCH-L1 than NT-UCH-L1s, immunoblot was done in separate membrane and the border lines were drawn. (H) SN4741 cells were transiently transfected with various amounts of pcDNA3.1 NT-UCH-L1-myc expressing plasmid. Cells were divided into soluble and insoluble fractions and analyzed in non-reducing ((-) 2-ME) and reducing ((+) 2-ME) gels. Proteins were immunoblotted using anti-myc and anti-α-tubulin antibodies. (I) HeLa stable cells expressing UCH-L1-myc and NT-UCH-L1-myc were treated with DMSO or 10 µM MG132 for 16 h. Myc tagged proteins were probed with anti-myc and Alexa Flour 488 secondary antibody (green), mitochondria were stained with Mitotracker (red) and nucleus were stained with DAPI (blue). Cells were examined under confocal microscope.

We found that the levels of both transiently and stably expressed NT-UCH-L1 in cells are less than those of UCH-L1. Based on the HDX result and the lower level of NT-UCH-L1 than UCH-L1, we postulated that NT-UCH-L1 has more unstable structure resulting in faster degradation than UCH-L1. We estimated the life time of UCH-L1 and NT-UCH-L1 by inhibiting protein synthesis with cycloheximide (CHX) in HeLa cells expressing UCH-L1-myc, NT-UCH-L1-myc, and K15/157R NT-UCH-L1-myc, and assessing the time course of disappearance of the protein bands by immunoblotting. NT-UCH-L1-myc and K15/157R NT-UCH-L1-myc disappeared faster than UCH-L1 (Fig. 5A and 5B). NT-UCH-L1 and K15/157R NT-UCH-L1 has half-lives approximately 6 h and 3 h, respectively, while UCH-L1's half-life is longer than 24 h. Intriguingly, K15/157R NT-UCH-L1-myc, which is not ubiquitinated, disappeared more readily than NT-UCH-L1. This indicates that NT-UCH-L1 has shorter life than UCH-L1 and monoubiquitination increases the stability of NT-UCH-L1. In case of UCH-L1, inhibition of monoubiquitination did not affect its life span (Fig. S3B).

To understand how NT-UCH-L1 is degraded in normal condition, we compared NT-UCH-L1 levels in cells treated with MG132, NH_4_Cl, and 3MA (Fig. 5C and 5D). We found that only MG132 induced the accumulation of NT-UCH-L1 and its K15/157R mutant in HeLa cells suggesting that in steady state as well as CCCP treated condition, NT-UCH-L1 is readily degraded by ubiquitin-proteasome system.

### NT-UCH-L1 is aggregation prone

UCH-L1 is a major component of the protein aggregates called Lewy bodies found in the brains of PD patients [2]. We examined the aggregation characteristics of NT-UCH-L1 accumulating in cells. Cells were treated with MG132 and separated into detergent soluble (S) and insoluble (I) fractions (Fig. 5E and 5F). NT-UCH-L1 and its K15/157R mutant significantly accumulated in the insoluble fraction, while negligible amounts of UCH-L1 were detected in the insoluble fraction after MG132 treatment. Also, only ubiquitination free NT-UCH-L1 was present in the insoluble fraction after MG132 treatment (Fig. 5E and 5F, middle panel). When we examined the insoluble fraction in a dopaminergic neuronal progenitor cell line SN4741, we found more than 20% of the transiently expressed NT-UCH-L1 and K15/157R NT-UCH-L1 in insoluble fractions, even without MG132 treatment, while most of the UCH-L1 was found in the soluble fraction (Fig. 5G). With increasing expression of NT-UCH-L1, its insoluble fraction increased (Fig. 5H, top panel). When 0.5 and 1.5 µg of NT-UCH-L1-myc expressing DNA were used for transient transfection, most of NT-UCH-L1 was in the soluble fraction. However, a part of NT-UCH-L1 expressed from 3 µg of DNA was in the insoluble fraction. This suggests that there is a threshold for the level of soluble NT-UCH-L1, and above this threshold, NT-UCH-L1 becomes insoluble in cells. We confirmed the tendency of NT-UCH-L1 to aggregate and become insoluble in cells, by examining the cells under confocal microscopy. NT-UCH-L1, not UCH-L1, was found aggregated in response to MG132 treatment (Fig. 5I). These results demonstrate that NT-UCH-L1 is more aggregation-prone than UCH-L1.

### Cys90 and Cys132 of NT-UCH-L1 form disulfide bonds

**Figure 6:**
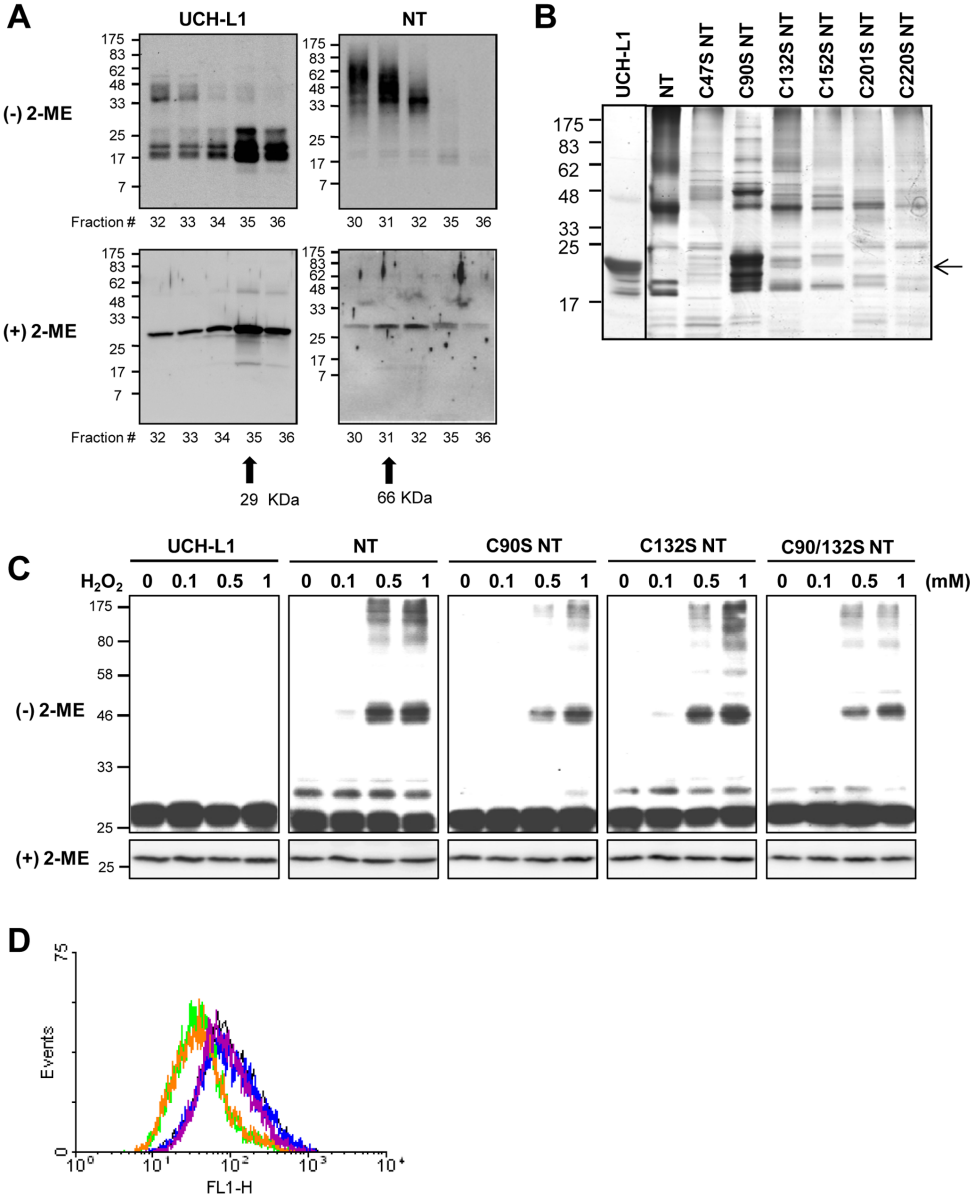
Disulfide bond formation and ROS lowering effect of NT-UCH-L1. (A) Recombinant UCH-L1 and NT-UCH-L1 were fractionated using FPLC. The fractions were resolved in non-reducing ((−) 2-ME) and reducing ((+) 2-ME) SDS-PAGE gels. UCH-L1 and NT-UCH-L1 were immunoblotted using anti-UCH-L1 antibody. (B) Recombinant UCH-L1, NT-UCH-L1, and its Cys mutants were analyzed in non-reducing SDS-PAGE gel and visualized by silver staining. Arrows indicate position of monomer UCH-L1 and NT-UCH-L1. (C) HeLa cells expressing UCH-L1-myc, NT-UCH-L1-myc, C90S NT-UCH-L1-myc, C132S NT-UCH-L1, and C90/132S NT-UCH-L1-myc were treated with various concentrations of H_2_O_2_ for 1 h and analyzed using non-reducing ((−) 2-ME) and reducing ((+) 2-ME) gels. UCH-L1s were immunoblotted using anti-myc antibody. (E) HeLa cells stably expressing UCH-L1-myc, NT-UCH-L1-myc, K15/157R NT-UCH-L1-myc and C90/132S NT-UCH-L1-myc were stained with CM-H_2_DCFDA and analyzed cellular ROS level using FACS analyzer. Black, mock; blue, UCH-L1; green, NT-UCH-L1; orange, K15/157R NT-UCH-L1; magenta, C90/132S NT-UCH-L1.

To investigate the nature of the insoluble aggregates of NT-UCH-L1, the soluble and insoluble fractions were analyzed in reducing ((+) 2-ME) and non-reducing ((−) 2-ME) conditions (Fig. 5H). Some of the insoluble NT-UCH-L1 appeared to be crosslinked through disulfide bonds, as it was detected at a high molecular weight complex on non-reducing gel (Fig. 5H, lower panel, S-S NT), but not UCH-L1 (Fig. S3C, lower panel). To confirm that NT-UCH-L1 is able to form disulfide crosslinking by themselves, recombinant UCH-L1 and NT-UCH-L1 were separated in gel filtration FPLC based on molecular weight. Fig. 6A shows that UCH-L1 mostly eluted as monomers (of about 29 kDa), while NT-UCH-L1 eluted as multimers larger than 66 kDa (Fig. 6A, upper panel). These high molecular weight NT-UCH-L1s dissociate into monomers with similar molecular weight with UCH-L1 under reducing condition ((+) 2-ME) (Fig. 6A, lower panel). This indicates that insoluble aggregates of NT-UCH-L1 contain intermolecular disulfide crosslinking.

The cysteine residues involved in the formation of insoluble aggregates, were identified by mutational and proteomic approaches. Under the non-reducing condition, NT-UCH-L1 having 6 Cys residues including Cys90 at its active site, formed multimers (Fig. 6B, lane 1). We performed mutagenesis of each 6 Cys to serine and examined their disulfide crosslinking formation. The C90S NT-UCH-L1 contained fewer multimers and more monomers of size around 25 kDa than other mutants (Fig. 6B, arrow). Disulfide crosslinking sites in NT-UCH-L1 multimer were also identified by peptide sequencing with nonoUPLC ESI-q-TOF MS/MS combined with the searching algorithm of disulfide site, DBond [54]. We detected many disulfide containing peptides (Table 1). Of the two surface cysteine residues, Cys132 and Cys152 [56], Cys132 formed disulfide bonds with all 6 Cys including itself. Based on these results, we generated C90S, C132S, and C90/132S mutants of NT-UCH-L1 and investigated whether they form less disulfide crosslinkings in response to oxidative stress. Cells were treated with H_2_O_2_ and the proteins were separated under both reducing and non-reducing conditions (Fig. 6C). UCH-L1 did not form disulfide bonded multimers, but NT-UCH-L1 did, in cells treated with 0.5 and 1 mM H_2_O_2_. C90S NT-UCH-L1 formed fewer multimers than NT-UCH-L1 or C132S NT-UCH-L1. This indicates that NT-UCH-L1 forms disulfide bonds mainly through Cys90 in response to oxidative stress.

**Table 1:** Disulfide bonds identified in aggregated NT-UCH-L1 by nanoUPLC-ESI-q-TOF tandem MS combining DBond searching algorithm.

Disulfide bonds	Peptides with disulfide bonds
	
C90–C132	QTIGNS**C**GTIGLIHAVANNQDK - **C**FEK
C90–C201	QTIGNS**C**GTIGLIHAVANNQDK - V**C**R
C90–C220	QTIGNS**C**GTIGLIHAVANNQDK - FSAVAL**C**K
C132–C132	**C**FEK – **C**FEK
C132–C152	**C**FEK - NEAIQAAHDAVAQEGQ**C**R
C132–C201	AKCFEK – VCR
C132–C220	**C**FEK - FSAVAL**C**K
	AK**C**FEK - FSAVAL**C**K
C152–C152	NEAIQAAHDAVAQEGQ**C**R - NEAIQAAHDAVAQEGQ**C**R
C152–C201	NEAIQAAHDAVAQEGQ**C**R - V**C**R
C152–C220	NEAIQAAHDAVAQEGQ**C**R - FSAVAL**C**K
C201–C220	V**C**R – FSAVAL**C**K
	V**C**REFTER - FSAVAL**C**K

### NT-UCH-L1 reduces cellular ROS levels

The mitochondrial localization of NT-UCH-L1 prompted us to speculate on its possible function in mitochondria. Since mitochondria are part of the major intracellular ROS generation machinery and NT-UCH-L1 is sensitive to oxidative stress, we examined the possible role of NT-UCH-L1 in controlling ROS levels. We assessed cellular ROS levels by measuring fluorescence generated by ROS after loading the cells with CM-H_2_DCFDA. Expression of NT-UCH-L1 and K15/157R NT-UCH-L1 resulted in decreased cellular ROS levels by approximately 2-fold, compared to the expression of mock and UCH-L1 (Fig. 6D). However, C90/132S NT-UCH-L1 did not lower ROS levels. These results suggest a relationship between disulfide formation in NT-UCH-L1 and cellular ROS levels.

### NT-UCH-L1 protects cells from H_2_O_2_, rotenone, and CCCP induced damage

**Figure 7:**
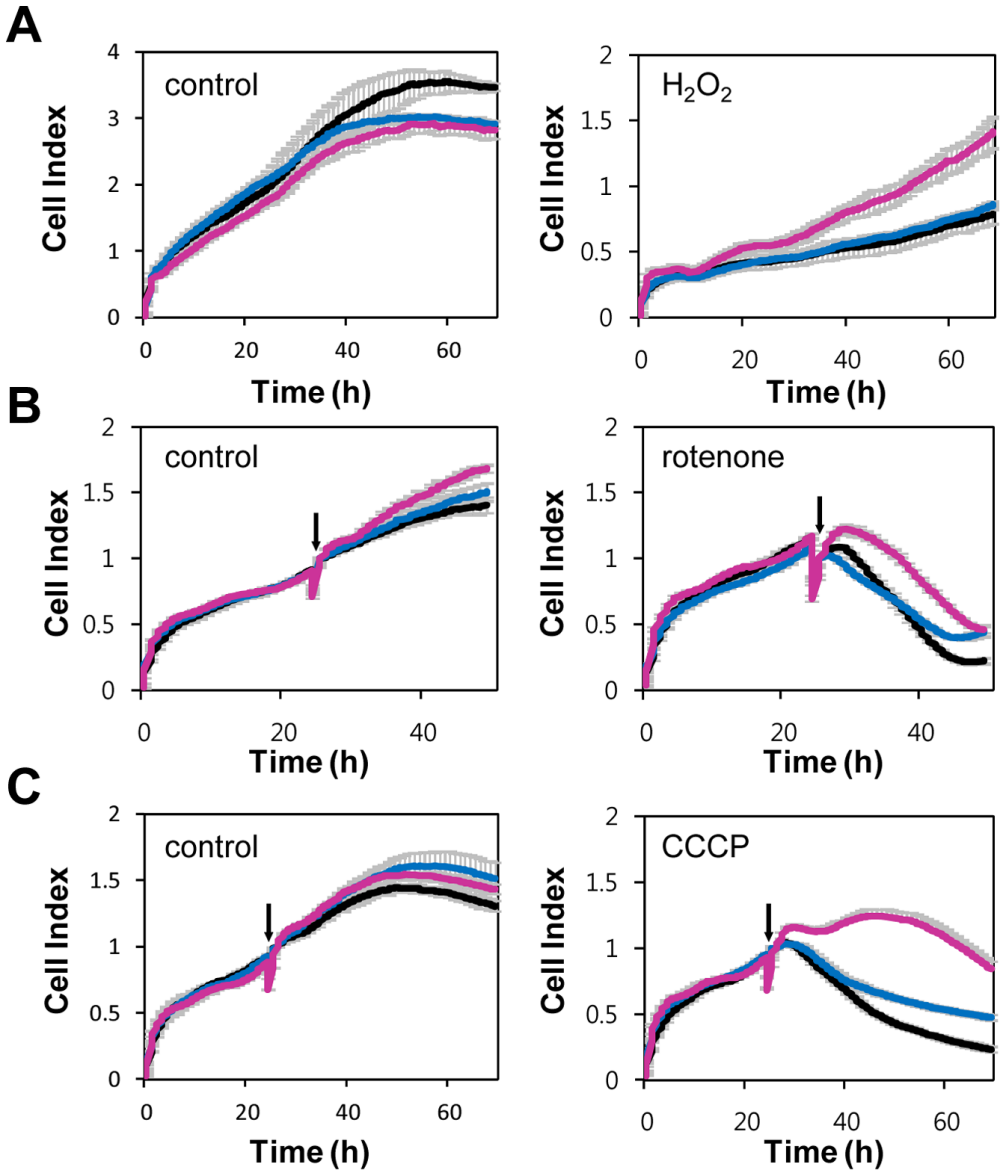
Protective effect of NT-UCH-L1 on PD related toxin induced damage. HeLa cells stably expressing UCH-L1-myc and NT-UCH-L1-myc were treated with 2 mM H_2_O_2_ for 1 h before plating cells (A) or 500 nM rotenone (B) and 25 µM CCCP (C) from the time indicated by arrow to the end of the experiment. Cell growth was monitored using real-time cell analyzer. Values represent mean of triplicates ± s.d. Black, mock; blue, UCH-L1; magenta, NT-UCH-L1.

Oxidative damage such as the one produced with H_2_O_2_ induces cell death which is related to the onset of PD [57], [58]. We tested if NT-UCH-L1 functions in H_2_O_2_ induced cell death. Cells were treated with 2 mM H_2_O_2_ for 1 h and monitored cell growth using a real-time cell analyzer (Fig. 7A). We found that cells expressing NT-UCH-L1 but not UCH-L1 were resistant to H_2_O_2_-induced cell death. Accumulating evidences suggests that mitochondrial dysfunction is another contributor to the pathogenesis of PD. Reduced activity of the mitochondrial electron transfer chain in PD has been reported [59], [60]. Rotenone and CCCP are known to induce mitochondrial damage and cell death [31], [61], we therefore examined whether NT-UCH-L1 plays a role in rotenone and CCCP induced cell death. Cells were treated with rotenone and CCCP and monitored cell growth using a real-time cell analyzer (Fig. 7B and 7C). We found that cells expressing NT-UCH-L1, but not UCH-L1 were resistant to rotenone- and CCCP-induced cell death.

### Generation of human NT-UCH-L1-myc expressing transgenic mice

**Figure 8:**
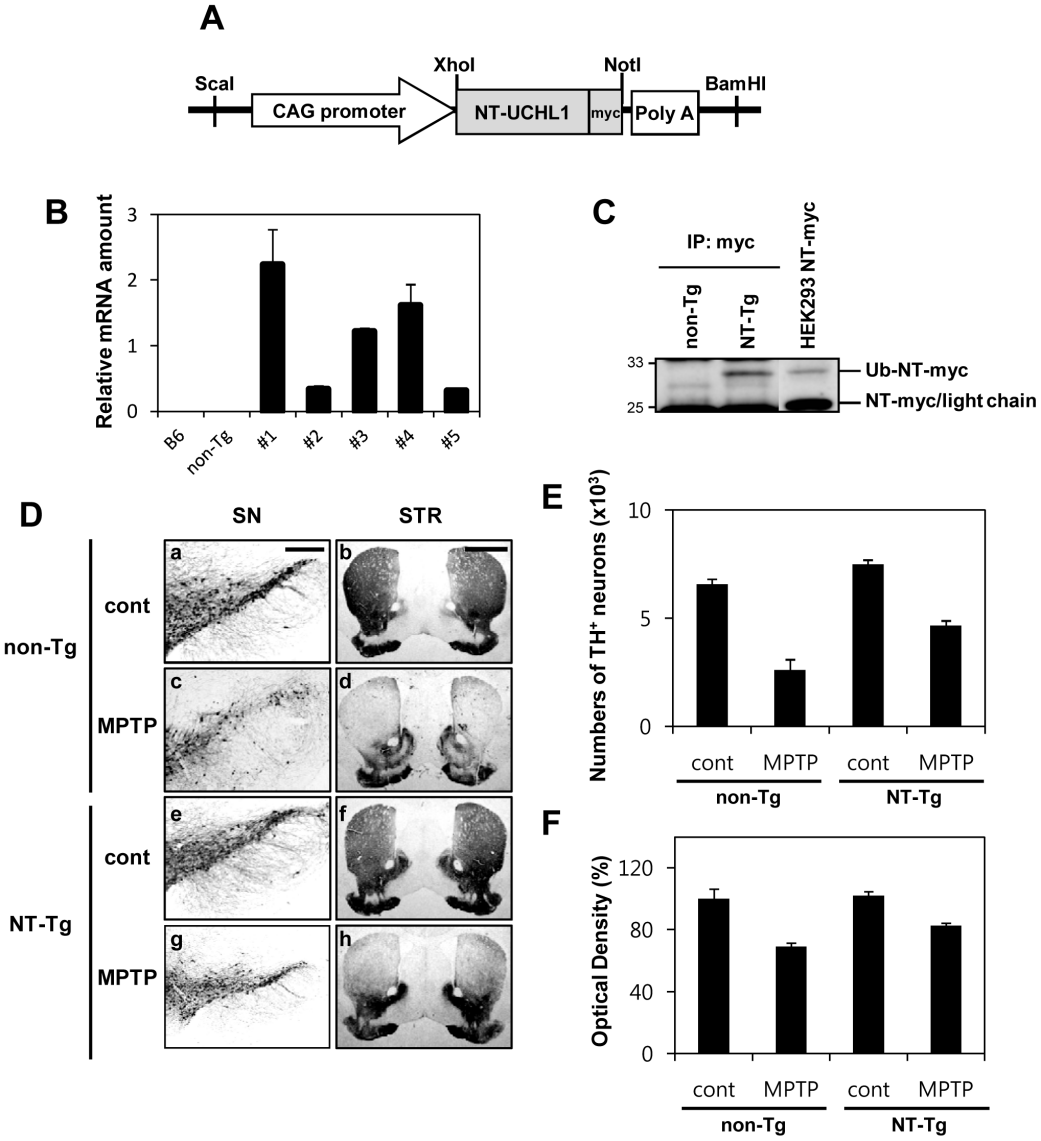
Generation of hNT-UCH-L1-myc transgenic mice and the protective role of NT-UCH-L1 in the MPTP model of Parkinson's disease. (A) NT-UCH-L1 was constructed under control of the CAG promoter. (B) Quantification of the mRNAs of the NT-UCH-L1-myc transgene from five transgenic mice lines using quantitative RT-PCR. (C) Immunoprecipitation analysis of hNT-UCH-L1-myc in control and NT-Tg mouse brain using anti-myc antibody. Immunoprecipitants were analyzed by immunoblotting method using anti-myc antibody. 3^rd^ land, cell lysate of HEK293 cells transiently expressing NT-UCH-L1-myc. (D) Immunohistochemical analysis showing neuroprotective effect of NT-UCH-L1 on nigrostriatal dopaminergic neurons. Mice in each group (non-Tg control (a, b), non-Tg MPTP (c, d), NT-UCH-L1 control (e, f) and NT-UCH-L1 MPTP (g, h)) were sacrificed 7 d after the last MPTP injection (c, d, g, h) or PBS as controls (a, b, e, f) and brain tissues were processed for tyrosine hydroxylase (TH) immunostaining in the substantia nigra pars compacta (SN, a, c, e, g) and striatum (STR, b, d, f, h). Scale bar, a, c, e, g, 100((m; b, d, f, h, 50((m. (E, F) The number of TH-positive cells in the SN (D) and optical density of TH-positive striatal fibers (D) are shown in graphs. n = 3 or 4 for each experimental group.

Finally, we addressed the question of whether the protective effects of NT-UCH-L1 against toxins seen in cell culture (Fig. 7) might also be manifested *in vivo*. We generated transgenic mice expressing human NT-UCH-L1 with myc tag (NT-Tg). We used a CAG promoter to drive expression of NT-UCH-L1 in transgenic mice (Fig. 8A). Germline transmission of NT-UCH-L1 was obtained in five independent NT-Tg lines and the levels of transgenic mRNA were assessed by quantitative RT-PCR (Fig. 8B). We chose the line #1 in which expression of NT-UCH-L1-myc mRNA was the highest for further analysis. We detected NT-UCH-L1-myc protein in mouse whole brain by immunoprecipitation and Western blot analysis (Fig. 8C, lane 2) co-migrating with the NT-UCH-L1 transiently expressed in HEK293 cells (Fig. 8C, lane 3). We confirmed that by silver staining and MS/MS to identify the immuprecipitated NT-UCH-L1 (Fig. S4A). We detected a faint silver stained band and identified a peptide of UCH-L1 only in the immunoprecipitant of NT-Tg but not in non-Tg sample.

### NT-UCH-L1 protects nigrostriatal dopaminergic neurons from the MPTP neurotoxicity in transgenic mice expressing human NT-UCH-L1

We then tested the effect of NT-UCH-L1 on the survival of DA neurons in the MPTP mouse model of PD. The brains of PBS or MPTP injected mice were removed and sectioned. The sections were immunostained for tyrosine hydroxylase (TH) to specifically detect DA neurons. As we reported previously [49]-[51], immunohistochemical analysis, stereological counts, and densitometric analyses revealed a 60% loss of TH-positive cell bodies in the substantia nigra pars compacta (SNpc) (Fig. 8D-c and E), and a 31% loss in TH-positive fibers in the striatum (STR) (Fig. 8D-d and F) in MPTP-injected non-Tg mice, compared with those in PBS control of non-Tg mice (Fig. 8D-a, b, E, and F). In contrast, the number of TH-positive cell bodies in the SNpc (Fig. 8D-g and E) and the density of TH-positive fibers in the STR (Fig. 8D-h and F) were significantly higher in the MPTP-treated NT-Tg mice, compared with MPTP-treated non-Tg mice (Fig. 8D-c, d, E, and F). When we stained cells with Cresyl violet stain and Cresyl violet/TH double staining, we also observed higher survival rates of SNpc cells in MPTP treated NT-Tg mice than in non-Tg mice (Fig. S4B and S4C). These findings suggest that NT-UCH-L1 promotes the survival of nigrostriatal DA neruons in the MPTP model of PD.

## Discussion

In this study, we found that a variant of UCH-L1 lacking 11 N-terminal amino acids, that we designated NT-UCH-L1, exists in lung cancer and neuronal cells, and in brain tissue. NT-UCH-L1 differs from UCH-L1 in its tertiary structure, in its lack of deubiquitinating activity, lack of ability to increase cell migration, in its greater tendency to be monoubiquitinated and readily aggregated.

Protein isoforms from one gene are produced by alternative splicing, alternative promoters, or alternative translation initiation sites. The 11 N-terminal amino acids missing in NT-UCH-L1, corresponds to exon 1 of the *UCH-L1* gene (*PARK5*). The exon 1-deleted mRNA has been reported to be expressed originally in a protein given a different name, PGP9.5 (NCBI accession number X04741) [62]. Thus, NT-UCH-L1 appears to be formed by translation from an alternatively spliced mRNA. Compared to alternative splicing and alternative promoters, alternative translation initiation was less studied. Recently, alternative translation start sites within a single transcript have been paid more attention [63]. As NT-UCH-L1 has a starting codon and an additional downstream in-frame AUG codon, alternative translation initiation from the 12^th^ methionine may also possible. Several examples including human insulin-degrading enzyme [64], human neuropeptide Y [65], *AtLIG1* gene in *Arabidopsis thaliana* [66], rat ornithine decarboxylase-enzyme [67], are reported as having alternative start codons and the isoforms showed distinguished subcellular localizations [68].

To compare the amounts of UCH-L1 and NT-UCH-L1 in mouse brain tissue and cell lines, we separated them in 2D-PAGE and probed with two different kinds of antibodies; one for both UCH-L1 and NT-UCH-L1 and the other only for UCH-L1. Significant amounts of NT-UCH-L1 exist in mouse brain tissue, neuronal cells, and lung cancer cells (Fig. 1A and 1B).

We further compared UCH-L1 and NT-UCH-L1 with regard to their structures, stability, modifications, localizations, and biological functions. NT-UCH-L1 did not exhibit the ubiquitin hydrolase activity of UCH-L1 (Fig. 1D and S1C). In HDX data (Figure 2C), the peptide containing active site Cys90 of NT-UCH-L1 was less deuterium exchanged than UCH-L1. It might explain different enzyme activities of UCH-L1 and NT-UCH-L1. In addition, UCH-L3 sharing considerable homology with UCH-L1 (52% amino acid identity) was postulated that it contacts basic residues located on ubiquitin with its acidic residues (Glu10, Glu14, Asp33, Glu219) [69]. All of the acidic residues are conserved in UCH-L1 and among them, Glu10 and Glu14 are missing in NT-UCH-L1. This suggests that N-terminal 11-peptide is required for catalytic activity in addition to active site. Employing HDX mass spectrometry, we found that NT-UCH-L1 has a more flexible structure than UCH-L1. During the preparation of recombinant NT-UCH-L1, it forms visible protein aggregates more easily than UCH-L1. This indicates that NT-UCH-L1 has unfolded structure similar to another PD causing gene product α-synuclein [70]. Alpha-synuclein is typically unfolded and can form various kinds of oligomeric structures such as pore forming structure, fibrils, and amorphous aggregates [19], [20]. As shown in Fig. 5H lower panel, not all of the NT-UCH-L1 in the insoluble fraction forms disulfide crosslinkings and there still exists disulfide linkage free NT-UCH-L1 (arrow head in the lane 8). It is possible that NT-UCH-L1 also has diverse kinds of muntimer forms and studying oligomeric structures of NT-UCH-L1 in detail would be interesting.

Protein monoubiquitination plays roles in DNA repair, histone regulation, gene expression, and receptor endocytosis [25]. In this study, we showed another function of monoubiquitination: it also changes subcellular localization and solubility of a protein. We showed that Ub-NT-UCH-L1 localized in the cytosol and ubiquitination free NT-UCH-L1 mainly localized in the mitochondria. In addition, we showed monoubiquitination free form of NT-UCH-L1 but not Ub-NT-UCH-L1 became insoluble when accumulated in cells. As monoubiquitination free form of NT-UCH-L1 localized in mitochondria and it became more insoluble in MG132 treated cells, it is possible that NT-UCH-L1 forms insoluble particle in mitochondria.

NT-UCH-L1 was easily oxidized by H_2_O_2_ treatments, forming inter-molecular disulfide bonds and cells expressing NT-UCH-L1 resist H_2_O_2_ induced cell death. This is similar to the action of DJ-1 under oxidizing environment. Cysteines in DJ-1 are easily oxidized in response to ROS and play antioxidant roles [71]–[73]. Further studies of the relationships among disulfide bond formations, lowering cellular ROS levels, and protection of cells from oxidative stress by NT-UCH-L1, would be informative.

**Figure 9:**
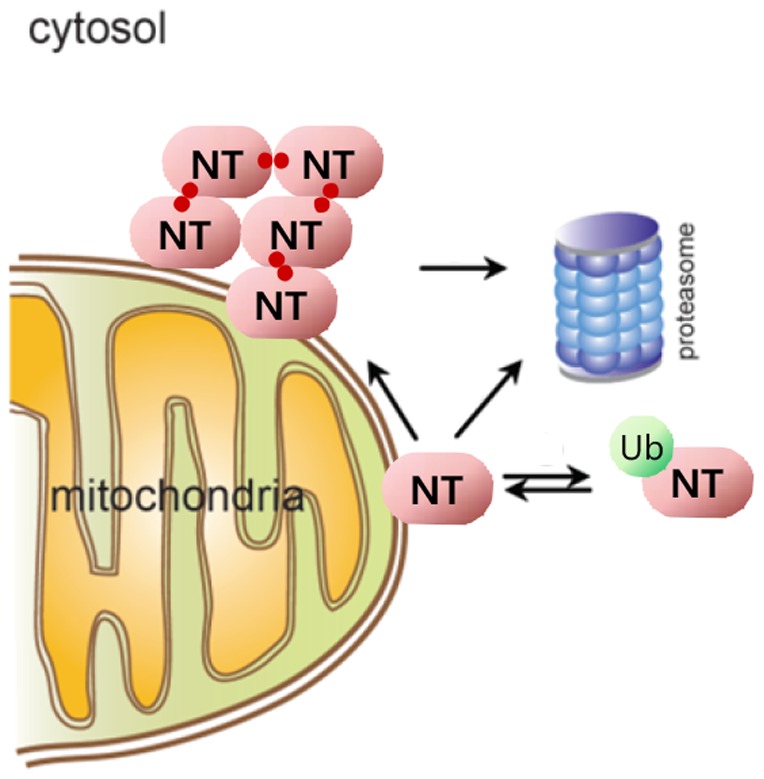
A suggested mechanism for molecular regulation of NT-UCH-L1. NT, NT-UCH-L1; Ub, monoubiquitin; two red circles, disulfide bond.

Based on our results, we suggest a molecular mechanism involving ubiqitination for the role of NT-UCH-L1 in protecting cells against stresses. This suggested mechanism is summarized as follows (Fig. 9). NT-UCH-L1 present in the mitochondria is in a state of equilibrium with Ub-NT-UCH-L1 present in the cytosol. When the amount of NT-UCH-L1 is increased above saturation level or when the cells are stressed, monoubiquitination free NT-UCH-L1 aggregates via intermolecular disulfide bonds mostly involving Cys90. Once NT-UCH-L1 becomes insoluble, it does not seem to go back to soluble fraction. The aggregates thus formed in the mitochondria are cleared by proteasome. NT-UCH-L1 is degraded also by proteasome in steady state.

This study suggests that NT-UCH-L1 has the potential for preventing neurotoxicity in PD. However, approaches to increasing NT-UCH-L1 levels in neurons to protect against mitochondrial or other damage might be hampered by thresholds for NT-UCH-L1 solubility, because NT-UCH-L1 forms insoluble protein aggregates once the concentration threshold is exceeded. This suggests that a fine control of the synthesis and degradation of NT-UCH-L1, is necessary for the maintenance of optimum levels of NT-UCH-L1. Currently, we do not know which factor(s) regulates the expression of NT-UCH-L1 and which E3 ubiquitin ligase promotes its monoubiquitination and degradation. Further studies are needed to shed light on qualitative and quantitative aspects of NT-UCH-L1's function in PD.

## Supporting Information

Figure S1 **N-terminal 11 amino acid truncated UCH-L1, NT-UCH-L1, doesn't have ubiquitin hydrolase enzyme activity.** (A) Isoforms and mutants of human UCH-L1 registered in NCBI. (B) UCH-L1 spots on the corresponding silver stained 2D gel were subjected to tryptic digestion and analyzed with MALDI-TOF MS. 505.2905 Da peak of ^12^MLNK^15^ peptide of NT-UCH-L1, instead of a peptide corresponding to the first 15 amino acids peak (1815.9144 Da) of UCH-L1, was detected and schematically represented. (c) Flag-UCH-L1 and Flag-NT-UCH-L1 transiently expressd in NCI-H157 cells were immunoprecipitated using anti-Flag antibody. Ubiquitn C-terminal hydrolase activities of the immunoprecipitates were measured using Ub-AMC as a substrate. pFlag-CMV-2 empty vector transfected cells (closed circle) were compared to Flag-UCH-L1 or Flag-NT-UCH-L1 expressing cells (open circle). Fluorescence of released free AMC was monitored at 460 nm. (D) HeLa cells transiently expressing Flag-NT-UCH-L1 or Flag-UCH-L1 cells were subjected to migration assay using transwell coated with Matrigel^™^. After 24 h, the number of migrated cells in the lower chamber was counted. The expression of Flag-NT-UCH-L1 and Flag-UCH-L1 in HeLa cells were shown by Western blot analysis of same number of cells using anti-Flag antibody. The mean ± s.d. of three independent experiments is shown. *p<0.05.
(TIF)

Figure S2 **NCI-H157 cells were analyzed using 2D-PAGE and visualized by Western blot analysis using anti-UCH-L1 (a), anti-N-terminal peptide (against the peptide, ^1^MQLKPMEINPE^11^, b) antibodies (lower panels).**
(TIF)

Figure S3 (A) HeLa cells transiently expressing UCH-L1-myc, K15/157R UCH-L1-myc, and NT-UCH-L1-myc were stained with anti-myc and Alexa Fluor 488 secondary antibody (green). Mitochondria and nucleus were stained with Mitotracker (red) and DAPI (blue), respectively. Cells were visualized by confocal microscopy. Lower pannel is enlarged figure of the dotted rectangle region of each upper pannel. (B) HeLa cells transiently expressing UCH-L1-myc, K15/157R UCH-L1-myc, and NT-UCH-L1-myc were treated with 10 µg/mL cycloheximide for the indicated times and immunoblotted using anti-myc antibody. (c) SN4741 cells were transiently transfected with pcDNA3.1 UCH-L1-myc or NT-UCH-L1-myc expressing plasmid. Cells were divided into soluble and insoluble fractions and analyzed in non-reducing ((-) 2-ME) and reducing ((+) 2-ME) gels. Proteins were immunoblotted using anti-myc antibody.
(TIF)

Figure S4 (A) Immunoprecipitation analysis of hNT-UCH-L1-myc in control and NT-Tg mouse brain using anti-myc antibody. Immunoprecipitants were analyzed by silver staining. The band only detected in NT-Tg sample (arrow head) was cut and identified by peptide finger printing and MS spectrometry. We observed one peptide peak with M.W. 742.9290 which matches with the UCH-L1 sequence, ^66^QIEELKGQEVSPK^78^. (B, C) Mice in each group (non-Tg control (a, b), NT-Tg (c, d)) were sacrificed 7 d after the last MPTP injection (b, d) or PBS as controls (a, c). Brain tissues were processed for Nissl staining (blue) (B) and Nissl (blue) and TH (brown) double staining (C). Dotted lines indicate substantia nigra pars compacta. Insets, pictures with higher magnifications. Scale bars, 100 µm.
(TIF)

## References

[1] Kim HJ, Kim YM, Lim S, Nam YK, Jeong J, et al (2009) Ubiquitin C-terminal hydrolase-L1 is a key regulator of tumor cell invasion and metastasis. Oncogene 28: 117–127. 18820707 10.1038/onc.2008.364

[2] Wilkinson KD, Lee KM, Deshpande S, Duerksen-Hughes P, Boss JM, et al (1989) The neuron-specific protein PGP 9.5 is a ubiquitin carboxyl-terminal hydrolase. Science 246: 670–673. 2530630 10.1126/science.2530630

[3] Lowe J, McDermott H, Landon M, Mayer RJ, Wilkinson KD (1990) Ubiquitin carboxyl-terminal hydrolase (PGP 9.5) is selectively present in ubiquitinated inclusion bodies characteristic of human neurodegenerative diseases. J Pathol 161: 153–160. 2166150 10.1002/path.1711610210

[4] Leroy E, Boyer R, Auburger G, Leube B, Ulm G, et al (1998) The ubiquitin pathway in Parkinson's disease. Nature 395: 451–452. 9774100 10.1038/26652

[5] Powers ET, Morimoto RI, Dillin A, Kelly JW, Balch WE (2009) Biological and chemical approaches to diseases of proteostasis deficiency. Annu Rev Biochem 78: 959–991. 19298183 10.1146/annurev.biochem.052308.114844

[6] Hartl FU, Hayer-Hartl M (2009) Converging concepts of protein folding in vitro and in vivo. Nat Struct Mol Biol 16: 574–581. 19491934 10.1038/nsmb.1591

[7] Goldberg AL (2003) Protein degradation and protection against misfolded or damaged proteins. Nature 426: 895–899. 14685250 10.1038/nature02263

[8] Iwata A, Christianson JC, Bucci M, Ellerby LM, Nukina N, et al (2005) Increased susceptibility of cytoplasmic over nuclear polyglutamine aggregates to autophagic degradation. Proc Natl Acad Sci U S A 102: 13135–13140. 16141322 10.1073/pnas.0505801102 PMC1201602

[9] Braak H, Del Tredici K, Rub U, de Vos RA, Jansen Steur EN, et al (2003) Staging of brain pathology related to sporadic Parkinson's disease. Neurobiol Aging 24: 197–211. 12498954 10.1016/s0197-4580(02)00065-9

[10] Cookson MR, Hardy J, Lewis PA (2008) Genetic neuropathology of Parkinson's disease. Int J Clin Exp Pathol 1: 217–231. 18784814 PMC2480564

[11] Tsika E, Moysidou M, Guo J, Cushman M, Gannon P, et al (2010) Distinct region-specific alpha-synuclein oligomers in A53T transgenic mice: implications for neurodegeneration. J Neurosci 30: 3409–3418. 20203200 10.1523/JNEUROSCI.4977-09.2010 PMC2844128

[12] Mazzulli JR, Xu YH, Sun Y, Knight AL, McLean PJ, et al (2011) Gaucher disease glucocerebrosidase and alpha-synuclein form a bidirectional pathogenic loop in synucleinopathies. Cell 146: 37–52. 21700325 10.1016/j.cell.2011.06.001 PMC3132082

[13] Forno LS (1969) Concentric hyalin intraneuronal inclusions of Lewy type in the brains of elderly persons (50 incidental cases): relationship to parkinsonism. J Am Geriatr Soc 17: 557–575. 4182529 10.1111/j.1532-5415.1969.tb01316.x

[14] Saito Y, Ruberu NN, Sawabe M, Arai T, Kazama H, et al (2004) Lewy body-related alpha-synucleinopathy in aging. J Neuropathol Exp Neurol 63: 742–749. 15290899 10.1093/jnen/63.7.742

[15] Dawson TM, Ko HS, Dawson VL (2012) Genetic animal models of Parkinson's disease. Neuron 66: 646–661. 10.1016/j.neuron.2010.04.034 PMC2917798 20547124

[16] Parkkinen L, Pirttila T, Tervahauta M, Alafuzoff I (2005) Widespread and abundant alpha-synuclein pathology in a neurologically unimpaired subject. Neuropathology 25: 304–314. 16382779 10.1111/j.1440-1789.2005.00644.x

[17] Tanaka M, Kim YM, Lee G, Junn E, Iwatsubo T, et al (2004) Aggresomes formed by alpha-synuclein and synphilin-1 are cytoprotective. J Biol Chem 279: 4625–4631. 14627698 10.1074/jbc.M310994200

[18] Tompkins MM, Hill WD (1997) Contribution of somal Lewy bodies to neuronal death. Brain Res 775: 24–29. 9439824 10.1016/s0006-8993(97)00874-3

[19] Goldberg MS, Lansbury PT Jr (2000) Is there a cause-and-effect relationship between alpha-synuclein fibrillization and Parkinson's disease? Nat Cell Biol 2: E115–119. 10878819 10.1038/35017124

[20] Uversky VN (2007) Neuropathology, biochemistry, and biophysics of alpha-synuclein aggregation. J Neurochem 103: 17–37. 17623039 10.1111/j.1471-4159.2007.04764.x

[21] Oueslati A, Fournier M, Lashuel HA (2010) Role of post-translational modifications in modulating the structure, function and toxicity of alpha-synuclein: implications for Parkinson's disease pathogenesis and therapies. Prog Brain Res 183: 115–145. 20696318 10.1016/S0079-6123(10)83007-9

[22] Kabuta T, Setsuie R, Mitsui T, Kinugawa A, Sakurai M, et al (2008) Aberrant molecular properties shared by familial Parkinson's disease-associated mutant UCH-L1 and carbonyl-modified UCH-L1. Hum Mol Genet 17: 1482–1496. 18250096 10.1093/hmg/ddn037

[23] Koharudin LM, Liu H, Di Maio R, Kodali RB, Graham SH, et al (2010) Cyclopentenone prostaglandin-induced unfolding and aggregation of the Parkinson disease-associated UCH-L1. Proc Natl Acad Sci U S A 107: 6835–6840. 20231490 10.1073/pnas.1002295107 PMC2872412

[24] Meray RK, Lansbury PT Jr (2007) Reversible monoubiquitination regulates the Parkinson disease-associated ubiquitin hydrolase UCH-L1. J Biol Chem 282: 10567–10575. 17259170 10.1074/jbc.M611153200

[25] Hicke L (2001) Protein regulation by monoubiquitin. Nat Rev Mol Cell Biol 2: 195–201. 11265249 10.1038/35056583

[26] Schapira AH, Cooper JM, Dexter D, Clark JB, Jenner P, et al (1990) Mitochondrial complex I deficiency in Parkinson's disease. J Neurochem 54: 823–827. 2154550 10.1111/j.1471-4159.1990.tb02325.x

[27] Hao LY, Giasson BI, Bonini NM (2010) DJ-1 is critical for mitochondrial function and rescues PINK1 loss of function. Proc Natl Acad Sci U S A 107: 9747–9752. 20457924 10.1073/pnas.0911175107 PMC2906840

[28] Yang Y, Gehrke S, Imai Y, Huang Z, Ouyang Y, et al (2006) Mitochondrial pathology and muscle and dopaminergic neuron degeneration caused by inactivation of Drosophila Pink1 is rescued by Parkin. Proc Natl Acad Sci U S A 103: 10793–10798. 16818890 10.1073/pnas.0602493103 PMC1502310

[29] Vives-Bauza C, Zhou C, Huang Y, Cui M, de Vries RL, et al (2010) PINK1-dependent recruitment of Parkin to mitochondria in mitophagy. Proc Natl Acad Sci U S A 107: 378–383. 19966284 10.1073/pnas.0911187107 PMC2806779

[30] Dauer W, Kholodilov N, Vila M, Trillat AC, Goodchild R, et al (2002) Resistance of alpha -synuclein null mice to the parkinsonian neurotoxin MPTP. Proc Natl Acad Sci U S A 99: 14524–14529. 12376616 10.1073/pnas.172514599 PMC137916

[31] Casarejos MJ, Menendez J, Solano RM, Rodriguez-Navarro JA, Garcia de Yebenes J, et al (2006) Susceptibility to rotenone is increased in neurons from parkin null mice and is reduced by minocycline. J Neurochem 97: 934–946. 16573651 10.1111/j.1471-4159.2006.03777.x

[32] Gautier CA, Kitada T, Shen J (2008) Loss of PINK1 causes mitochondrial functional defects and increased sensitivity to oxidative stress. Proc Natl Acad Sci U S A 105: 11364–11369. 18687901 10.1073/pnas.0802076105 PMC2516271

[33] Nguyen HN, Byers B, Cord B, Shcheglovitov A, Byrne J, et al (2011) LRRK2 mutant iPSC-derived DA neurons demonstrate increased susceptibility to oxidative stress. Cell Stem Cell 8: 267–280. 21362567 10.1016/j.stem.2011.01.013 PMC3578553

[34] Jenner P (2003) Oxidative stress in Parkinson's disease. Ann Neurol 53 Suppl 3S26–36 discussion S36–28. 12666096 10.1002/ana.10483

[35] Richardson JR, Quan Y, Sherer TB, Greenamyre JT, Miller GW (2005) Paraquat neurotoxicity is distinct from that of MPTP and rotenone. Toxicol Sci 88: 193–201. 16141438 10.1093/toxsci/kfi304

[36] Callio J, Oury TD, Chu CT (2005) Manganese superoxide dismutase protects against 6-hydroxydopamine injury in mouse brains. J Biol Chem 280: 18536–18542. 15755737 10.1074/jbc.M413224200 PMC1885201

[37] Vila M, Przedborski S (2003) Targeting programmed cell death in neurodegenerative diseases. Nat Rev Neurosci 4: 365–375. 12728264 10.1038/nrn1100

[38] Perier C, Bove J, Vila M, Przedborski S (2003) The rotenone model of Parkinson's disease. Trends Neurosci 26: 345–346. 12850429 10.1016/S0166-2236(03)00144-9

[39] Spector A, Roy D (1978) Disulfide-linked high molecular weight protein associated with human cataract. Proc Natl Acad Sci U S A 75: 3244–3248. 277922 10.1073/pnas.75.7.3244 PMC392751

[40] Deng HX, Shi Y, Furukawa Y, Zhai H, Fu R, et al (2006) Conversion to the amyotrophic lateral sclerosis phenotype is associated with intermolecular linked insoluble aggregates of SOD1 in mitochondria. Proc Natl Acad Sci U S A 103: 7142–7147. 16636275 10.1073/pnas.0602046103 PMC1447523

[41] Jeong J, Jung Y, Na S, Lee E, Kim MS, et al (2011) Novel oxidative modifications in redox-active cysteine residues. Mol Cell Proteomics 10: M110 000513. 10.1074/mcp.M110.000513 PMC3047142 21148632

[42] Dull T, Zufferey R, Kelly M, Mandel RJ, Nguyen M, et al (1998) A third-generation lentivirus vector with a conditional packaging system. J Virol 72: 8463–8471. 9765382 10.1128/jvi.72.11.8463-8471.1998 PMC110254

[43] Lee T, Hoofnagle AN, Resing KA, Ahn NG (2005) Hydrogen exchange solvent protection by an ATP analogue reveals conformational changes in ERK2 upon activation. J Mol Biol 353: 600–612. 16185715 10.1016/j.jmb.2005.08.029

[44] Kim MS, Jeong J, Shin DH, Lee KJ (2013) Structure of Nm23-H1 under oxidative conditions. Acta Crystallogr D Biol Crystallogr 69: 669–680. 23519676 10.1107/S0907444913001194

[45] Seo J, Jeong J, Kim YM, Hwang N, Paek E, et al (2008) Strategy for comprehensive identification of post-translational modifications in cellular proteins, including low abundant modifications: application to glyceraldehyde-3-phosphate dehydrogenase. J Proteome Res 7: 587–602. 18183946 10.1021/pr700657y

[46] Lee E, Jeong J, Kim SE, Song EJ, Kang SW, et al (2009) Multiple functions of Nm23-H1 are regulated by oxido-reduction system. PLoS one 4: e7949. 19956735 10.1371/journal.pone.0007949 PMC2776532

[47] Ardley HC, Scott GB, Rose SA, Tan NG, Markham AF, et al (2003) Inhibition of proteasomal activity causes inclusion formation in neuronal and non-neuronal cells overexpressing Parkin. Mol Biol Cell 14: 4541–4556. 12937272 10.1091/mbc.E03-02-0078 PMC266771

[48] Shim JH, Yoon SH, Kim KH, Han JY, Ha JY, et al.. (2011) The antioxidant Trolox helps recovery from the familial Parkinson's disease-specific mitochondrial deficits caused by PINK1- and DJ-1-deficiency in dopaminergic neuronal cells. Mitochondrion. 10.1016/j.mito.2011.05.013 21664494

[49] Chung YC, Kim SR, Jin BK (2010) Paroxetine prevents loss of nigrostriatal dopaminergic neurons by inhibiting brain inflammation and oxidative stress in an experimental model of Parkinson's disease. J Immunol 185: 1230–1237. 20566832 10.4049/jimmunol.1000208

[50] Chung YC, Kim SR, Park JY, Chung ES, Park KW, et al (2011) Fluoxetine prevents MPTP-induced loss of dopaminergic neurons by inhibiting microglial activation. Neuropharmacology 60: 963–974. 21288472 10.1016/j.neuropharm.2011.01.043

[51] Huh SH, Chung YC, Piao Y, Jin MY, Son HJ, et al (2011) Ethyl pyruvate rescues nigrostriatal dopaminergic neurons by regulating glial activation in a mouse model of Parkinson's disease. J Immunol 187: 960–969. 21685323 10.4049/jimmunol.1100009

[52] West MJ, Slomianka L, Gundersen HJ (1991) Unbiased stereological estimation of the total number of neurons in thesubdivisions of the rat hippocampus using the optical fractionator. Anat Rec 231: 482–497. 1793176 10.1002/ar.1092310411

[53] Osaka H, Wang YL, Takada K, Takizawa S, Setsuie R, et al (2003) Ubiquitin carboxy-terminal hydrolase L1 binds to and stabilizes monoubiquitin in neuron. Hum Mol Genet 12: 1945–1958. 12913066 10.1093/hmg/ddg211

[54] Bittencourt Rosas SL, Caballero OL, Dong SM, da Costa Carvalho Mda G, Sidransky D, et al (2001) Methylation status in the promoter region of the human PGP9.5 gene in cancer and normal tissues. Cancer Lett 170: 73–79. 11448537 10.1016/s0304-3835(01)00449-9

[55] Narendra D, Tanaka A, Suen DF, Youle RJ (2008) Parkin is recruited selectively to impaired mitochondria and promotes their autophagy. J Cell Biol 183: 795–803. 19029340 10.1083/jcb.200809125 PMC2592826

[56] Das C, Hoang QQ, Kreinbring CA, Luchansky SJ, Meray RK, et al (2006) Structural basis for conformational plasticity of the Parkinson's disease-associated ubiquitin hydrolase UCH-L1. Proc Natl Acad Sci U S A 103: 4675–4680. 16537382 10.1073/pnas.0510403103 PMC1450230

[57] Heikkila R, Cohen G (1971) Inhibition of biogenic amine uptake by hydrogen peroxide: a mechanism for toxic effects of 6-hydroxydopamine. Science 172: 1257–1258. 5576164 10.1126/science.172.3989.1257

[58] Lotharius J, O′Malley KL (2000) The parkinsonism-inducing drug 1-methyl-4-phenylpyridinium triggers intracellular dopamine oxidation. A novel mechanism of toxicity. J Biol Chem 275: 38581–38588. 10969076 10.1074/jbc.M005385200

[59] Parker WD Jr, Boyson SJ, Parks JK (1989) Abnormalities of the electron transport chain in idiopathic Parkinson's disease. Ann Neurol 26: 719–723. 2557792 10.1002/ana.410260606

[60] Schapira AH, Cooper JM, Dexter D, Jenner P, Clark JB, et al (1989) Mitochondrial complex I deficiency in Parkinson's disease. Lancet 1: 1269. 2566813 10.1016/s0140-6736(89)92366-0

[61] Parker WD Jr, Parks JK, Swerdlow RH (2008) Complex I deficiency in Parkinson's disease frontal cortex. Brain Res 1189: 215–218. 18061150 10.1016/j.brainres.2007.10.061 PMC2295283

[62] Day IN, Thompson RJ (1987) Molecular cloning of cDNA coding for human PGP 9.5 protein. A novel cytoplasmic marker for neurones and neuroendocrine cells. FEBS Lett 210: 157–160. 2947814 10.1016/0014-5793(87)81327-3

[63] Bazykin GA, Kochetov AV (2011) Alternative translation start sites are conserved in eukaryotic genomes. Nucleic Acids Res 39: 567–577. 20864444 10.1093/nar/gkq806 PMC3025576

[64] Leissring MA, Farris W, Wu X, Christodoulou DC, Haigis MC, et al (2004) Alternative translation initiation generates a novel isoform of insulin-degrading enzyme targeted to mitochondria. Biochem J 383: 439–446. 15285718 10.1042/BJ20041081 PMC1133736

[65] Kaipio K, Kallio J, Pesonen U (2005) Mitochondrial targeting signal in human neuropeptide Y gene. Biochem Biophys Res Commun 337: 633–640. 16199004 10.1016/j.bbrc.2005.09.093

[66] Sunderland PA, West CE, Waterworth WM, Bray CM (2004) Choice of a start codon in a single transcript determines DNA ligase 1 isoform production and intracellular targeting in Arabidopsis thaliana. Biochem Soc Trans 32: 614–616. 15270689 10.1042/BST0320614

[67] Gandre S, Bercovich Z, Kahana C (2003) Mitochondrial localization of antizyme is determined by context-dependent alternative utilization of two AUG initiation codons. Mitochondrion 2: 245–256. 16120325 10.1016/S1567-7249(02)00105-8

[68] Kochetov AV (2008) Alternative translation start sites and hidden coding potential of eukaryotic mRNAs. Bioessays 30: 683–691. 18536038 10.1002/bies.20771

[69] Wilkinson KD, Laleli-Sahin E, Urbauer J, Larsen CN, Shih GH, et al (1999) The binding site for UCH-L3 on ubiquitin: mutagenesis and NMR studies on the complex between ubiquitin and UCH-L3. J Mol Biol 291: 1067–1077. 10518943 10.1006/jmbi.1999.3038

[70] Valente EM, Abou-Sleiman PM, Caputo V, Muqit MM, Harvey K, et al (2004) Hereditary early-onset Parkinson's disease caused by mutations in PINK1. Science 304: 1158–1160. 15087508 10.1126/science.1096284

[71] Takahashi-Niki K, Niki T, Taira T, Iguchi-Ariga SM, Ariga H (2004) Reduced anti-oxidative stress activities of DJ-1 mutants found in Parkinson's disease patients. Biochem Biophys Res Commun 320: 389–397. 15219840 10.1016/j.bbrc.2004.05.187

[72] Taira T, Saito Y, Niki T, Iguchi-Ariga SM, Takahashi K, et al (2004) DJ-1 has a role in antioxidative stress to prevent cell death. EMBO Rep 5: 213–218. 14749723 10.1038/sj.embor.7400074 PMC1298985

[73] Canet-Aviles RM, Wilson MA, Miller DW, Ahmad R, McLendon C, et al (2004) The Parkinson's disease protein DJ-1 is neuroprotective due to cysteine-sulfinic acid-driven mitochondrial localization. Proc Natl Acad Sci U S A 101: 9103–9108. 15181200 10.1073/pnas.0402959101 PMC428480

