# Supplementary figures and images for: N-Terminal Truncated UCH-L1 Prevents Parkinson's Disease Associated Damage
Source: PLoS One. 2014 Jun 24;9(6):e99654. doi: 10.1371/journal.pone.0099654 (PMC4069018; doi:10.1371/journal.pone.0099654)

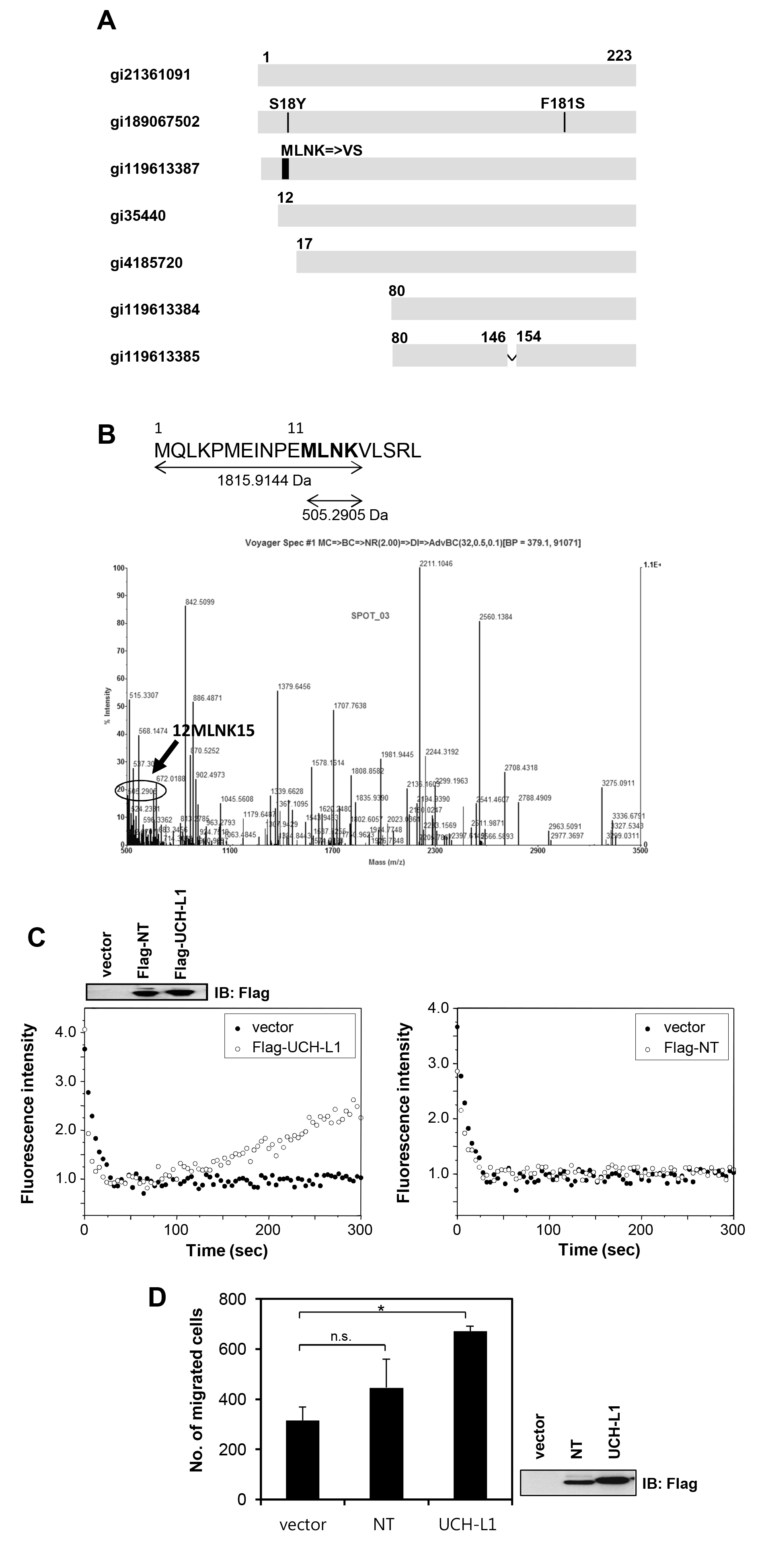

Supplement: Figure S1 — N-terminal 11 amino acid truncated UCH-L1, NT-UCH-L1, doesn't have ubiquitin hydrolase enzyme activity. (A) Isoforms and mutants of human UCH-L1 registered in NCBI. (B) UCH-L1 spots on the corresponding silver stained 2D gel were subjected to tryptic digestion and analyzed with MALDI-TOF MS. 505.2905 Da peak of 12MLNK15 peptide of NT-UCH-L1, instead of a peptide corresponding to the first 15 amino acids peak (1815.9144 Da) of UCH-L1, was detected and schematically represented. (c) Flag-UCH-L1 and Flag-NT-UCH-L1 transiently expressd in NCI-H157 cells were immunoprecipitated using anti-Flag antibody. Ubiquitn C-terminal hydrolase activities of the immunoprecipitates were measured using Ub-AMC as a substrate. pFlag-CMV-2 empty vector transfected cells (closed circle) were compared to Flag-UCH-L1 or Flag-NT-UCH-L1 expressing cells (open circle). Fluorescence of released free AMC was monitored at 460 nm. (D) HeLa cells transiently expressing Flag-NT-UCH-L1 or Flag-UCH-L1 cells were subjected to migration assay using transwell coated with Matrigel™. After 24 h, the number of migrated cells in the lower chamber was counted. The expression of Flag-NT-UCH-L1 and Flag-UCH-L1 in HeLa cells were shown by Western blot analysis of same number of cells using anti-Flag antibody. The mean ± s.d. of three independent experiments is shown. *p<0.05. (TIF) [file pone.0099654.s001.tif]

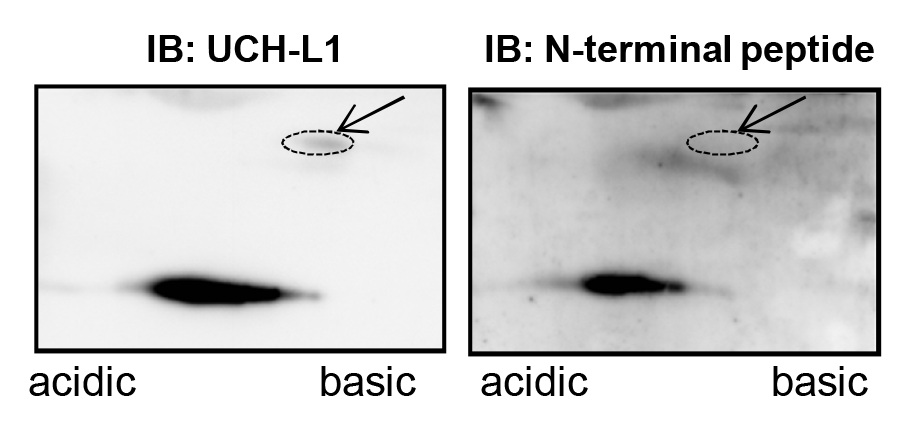

Supplement: Figure S2 — NCI-H157 cells were analyzed using 2D-PAGE and visualized by Western blot analysis using anti-UCH-L1 (a), anti-N-terminal peptide (against the peptide, 1MQLKPMEINPE11, b) antibodies (lower panels). (TIF) [file pone.0099654.s002.tif]

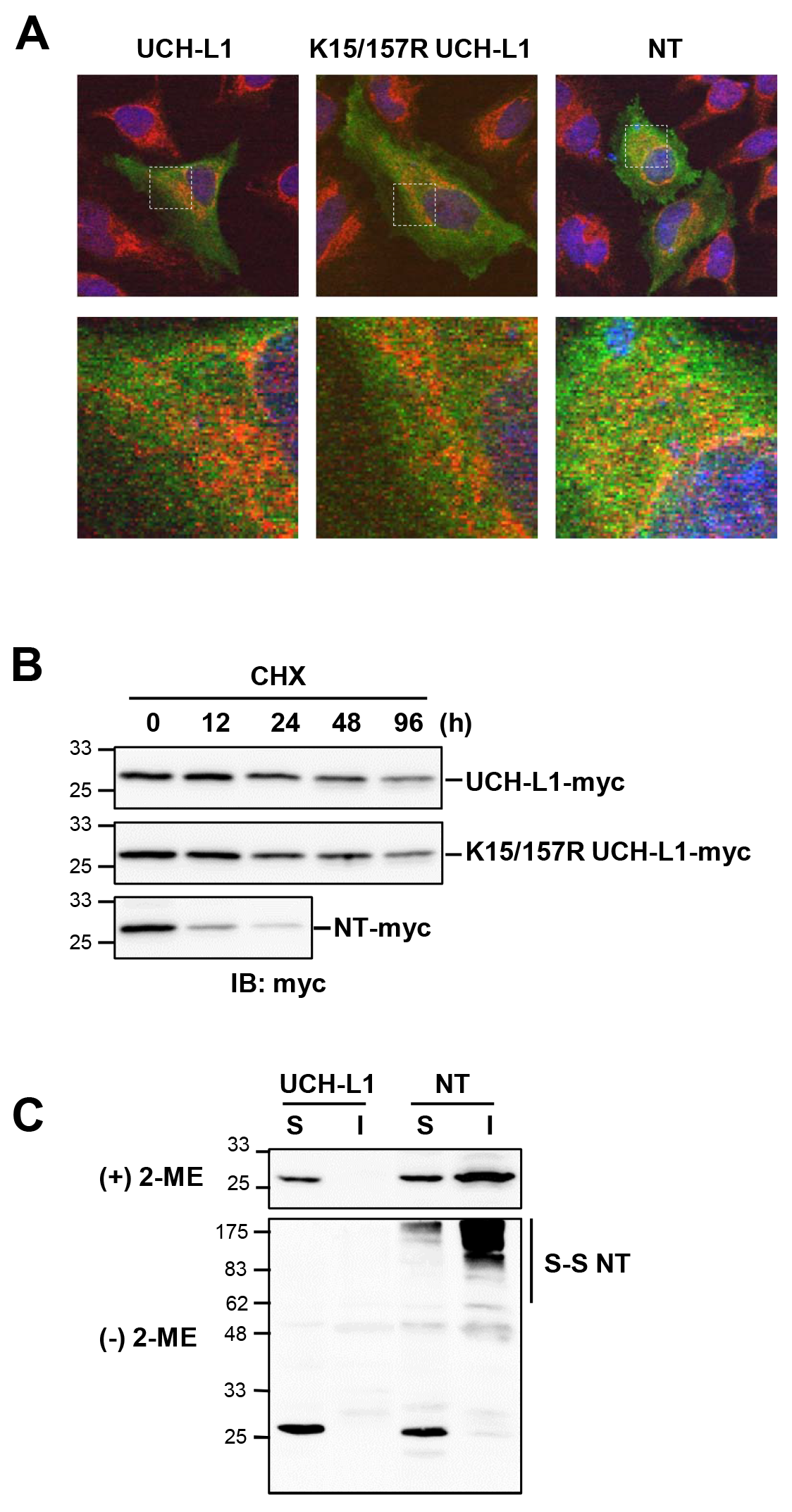

Supplement: Figure S3 — (A) HeLa cells transiently expressing UCH-L1-myc, K15/157R UCH-L1-myc, and NT-UCH-L1-myc were stained with anti-myc and Alexa Fluor 488 secondary antibody (green). Mitochondria and nucleus were stained with Mitotracker (red) and DAPI (blue), respectively. Cells were visualized by confocal microscopy. Lower pannel is enlarged figure of the dotted rectangle region of each upper pannel. (B) HeLa cells transiently expressing UCH-L1-myc, K15/157R UCH-L1-myc, and NT-UCH-L1-myc were treated with 10 µg/mL cycloheximide for the indicated times and immunoblotted using anti-myc antibody. (c) SN4741 cells were transiently transfected with pcDNA3.1 UCH-L1-myc or NT-UCH-L1-myc expressing plasmid. Cells were divided into soluble and insoluble fractions and analyzed in non-reducing ((-) 2-ME) and reducing ((+) 2-ME) gels. Proteins were immunoblotted using anti-myc antibody. (TIF) [file pone.0099654.s003.tif]

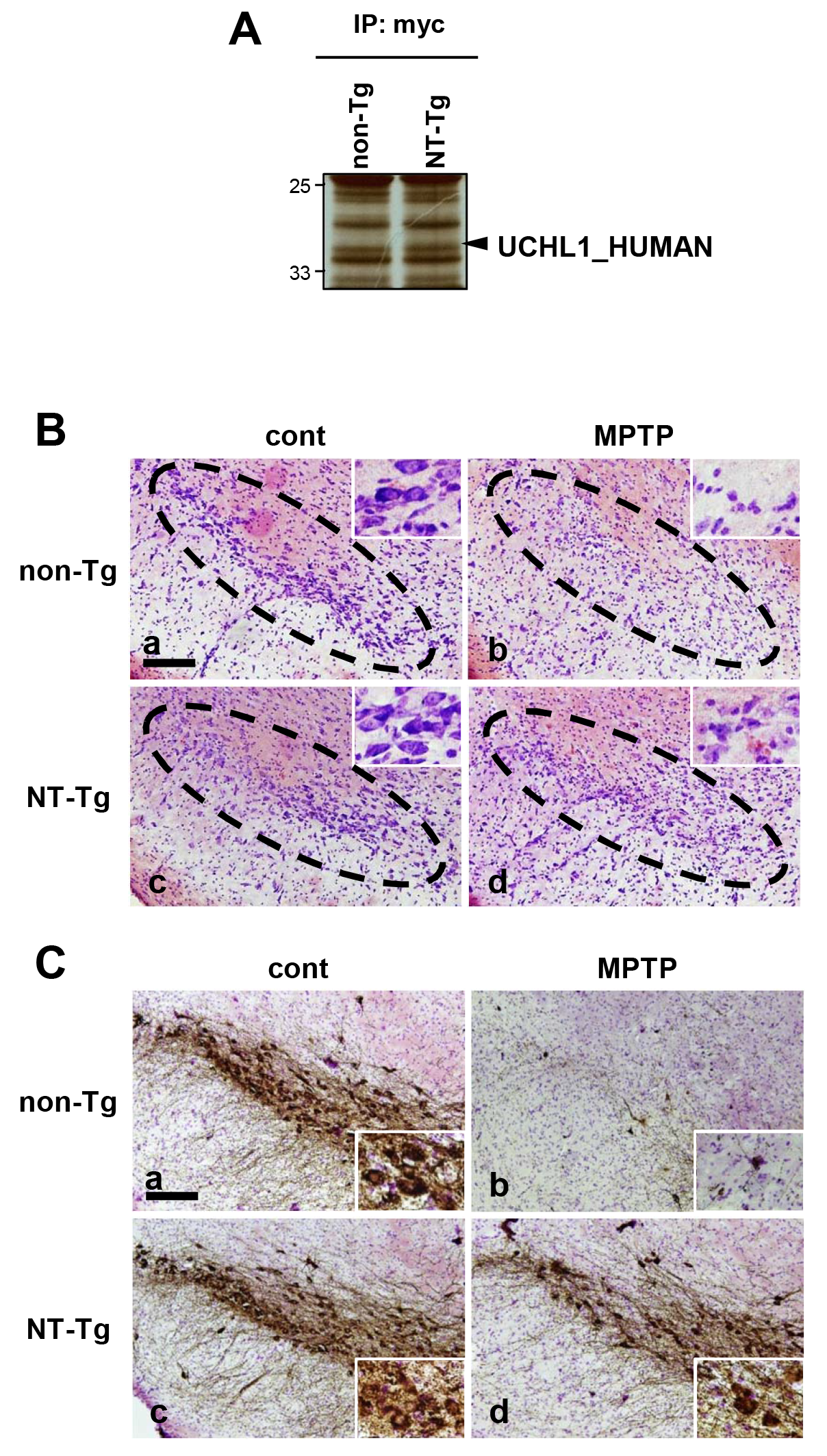

Supplement: Figure S4 — (A) Immunoprecipitation analysis of hNT-UCH-L1-myc in control and NT-Tg mouse brain using anti-myc antibody. Immunoprecipitants were analyzed by silver staining. The band only detected in NT-Tg sample (arrow head) was cut and identified by peptide finger printing and MS spectrometry. We observed one peptide peak with M.W. 742.9290 which matches with the UCH-L1 sequence, 66QIEELKGQEVSPK78. (B, C) Mice in each group (non-Tg control (a, b), NT-Tg (c, d)) were sacrificed 7 d after the last MPTP injection (b, d) or PBS as controls (a, c). Brain tissues were processed for Nissl staining (blue) (B) and Nissl (blue) and TH (brown) double staining (C). Dotted lines indicate substantia nigra pars compacta. Insets, pictures with higher magnifications. Scale bars, 100 µm. (TIF) [file pone.0099654.s004.tif]
